# A semi-automated, high throughput approach for *O*-glycosylation profiling of *in vitro* established cancer cell lines by MALDI-FT-ICR MS

**DOI:** 10.1007/s10719-021-10003-1

**Published:** 2021-07-20

**Authors:** Maximilianos Kotsias, Katarina Madunić, Simone Nicolardi, Radoslaw P. Kozak, Richard A. Gardner, Bas C. Jansen, Daniel I. R. Spencer, Manfred Wuhrer

**Affiliations:** 1 Ludger Ltd, Culham Science Centre, Abingdon, Oxfordshire UK; 2 Center for Proteomics and Metabolomics, Leiden University Medical Center, Leiden, Netherlands

## Abstract

The study of protein *O*-glycosylation is important in biological research as *O*-glycans have been reported to regulate a multitude of molecular and cell biology processes occurring in cancer. It is known that alterations in *O*-glycosylation are involved in the development and progression of cancer. Their easy accessibility makes *in vitro* established cell lines suitable and useful models for studying biological mechanisms in disease. However, the *O*-glycosylation analysis of large numbers of samples, as required in systems biology and biomarker discovery studies, is often challenging. In the present study, *O*-glycans from three human colorectal cancer cell lines and two human pancreatic cancer cell lines were released by semi-automated, high throughput reductive β-elimination and analysed using ultrahigh resolution MALDI-FT-ICR MS. Automated data integration and processing was performed using MassyTools, where the analyte was automatically included for relative quantitation based on a range of selection criteria including signal-to-noise ratio, mass error and isotopic pattern quality scores. A total of 126 *O*-glycan compositions, ranging from a single monosaccharide to large oligosaccharides exhibiting complex glycan motifs, were detected. The use of ultrahigh resolution MALDI-FTICR MS enabled glycan identification and quantitation in the matrix region of the spectrum. This approach has the potential to be used for *O*-glycosylation analysis of large numbers of samples, such as patient sample cohorts.

**Supplementary Information:**

The online version contains supplementary material available at 10.1007/s10719-021-10003-1.

## Introduction

Glycosylation is one of the most common co- and post-translational modifications of proteins [1–3]. *O*-glycans are known to be associated with many fundamental molecular and cell biology processes occurring in cancer, such as cell signaling and communication, tumour cell dissociation and invasion, angiogenesis, immune modulation and metastasis formation [3–8]. Additionally, alterations in *O*-glycosylation are associated with the development and progression of cancer, serving as important biomarkers and providing a set of specific targets for diagnosis and therapeutic intervention [9–13].

Glycosylation changes in malignant cells take a variety of forms such as the increased expression of incomplete or truncated glycans, and in rare cases the appearance of novel glycans [14]. Changes frequently observed in *O*-glycans include the overexpression of small or truncated glycans such as T, Tn, sialyl T, and sialyl Tn antigen, as well as the increased expression of sialyl Lewis antigens [3, 14].

Many different investigative approaches are currently employed for studying glycosylation changes in cancer. However, considering the limited availability of *ex vivo* tissue such as mucus, blood or epithelial cells, and conscious about the obstacles to overcome when it comes to performing animal studies, *in vitro* established cancer cell lines represent an attractive and relevant alternative and are widely used as model systems for studying the molecular mechanisms associated with cancer [15, 16]. *In vitro* cellular assays have supported the conclusion that glycan changes are critical to several aspects of tumour cell behavior [14, 16]. Hence, the development and/or optimization of glycoanalytical technologies that allow the precise and accurate analysis of glycosylation changes from these materials is of utmost importance.

However, *O*-glycosylation analysis of biological samples presents a number of challenges that need to be addressed. The laborious procedures that need to be performed for sample preparation and purification from contaminants originating from cell media and/or cellular debris, along with the high complexity of the glycan structures found in these samples can hinder the identification, characterization and quantitation of the glycan species [17]. Besides, the analysis of a large number of samples, often required for the identification of potential biomarkers and treatment targets, can prove a formidable task [18].

Notably, the release, recovery and analysis of *O*-glycans remains challenging due to the absence of a universal enzyme that can remove the full range of these compounds [19]. Therefore, their release often relies on chemical approaches [17, 19, 20]. One of the most common and reliable methods for the chemical removal of *O*-glycans is reductive β-elimination [20–22]. In this method *O*-glycans are released from the glycoprotein backbone and converted into the reduced alditol form using potassium or sodium hydroxide in combination with potassium or sodium borohydride [20, 22]. The reduction largely prevents oligosaccharide degradation under the alkaline release conditions. However, the complete prevention of this phenomenon remains difficult [22].

Recently, we proposed a semi-automated, high-throughput adaptation of this approach for *O*-glycosylation analysis of glycoproteins [20]. Considering recent advances with regards to high-throughput analyses, it is now clear that the development, optimization and routine employment of automated workflows for sample preparation, measurement, data curation and analysis is necessary for reducing the time required to prepare and measure *O*-glycan samples, allowing for accurate glycosylation analysis of larger samples sets and thus accelerating the detection of specific *O*-glycosylation changes.

With regards to glycan characterization, matrix-assisted laser desorption/ionization-mass spectrometry (MALDI-MS) analysis, commonly performed on a time-of-flight (TOF) mass analyzer, represents one of the most rapid approaches for glycan analysis [23]. However, *O*-linked glycans can vary vastly in size, ranging from a single *N*-acetylgalactosamine (GalNAc) to large oligosaccharides exhibiting complex glycan motifs. Therefore, higher resolving power is needed in order to distinguish small *O*-glycan structures from the signals of potentially interfering ion species such as MALDI matrix signals. The required higher resolving power and mass accuracy can be achieved by using Fourier Transform Ion Cyclotron Resonance Mass Spectrometers (FT-ICR MS) for detailed glycosylation analysis of complex samples [24, 25]. The use of this technology can enable the analysis of glycans in the MALDI matrix region of the spectrum as well as facilitate the differentiation of isobaric glycan species thanks to its sub-ppm mass measurement precision. Furthermore, the high mass accuracy over a large *m/z* range supports glycan identification [26].

Here we have optimized and applied the semi-automated, high-throughput reductive β-elimination workflow, coupled to ultrahigh resolution MALDI-FT-ICR MS for detailed *O*-glycosylation analysis of *in vitro* established cell lines. We used mucin from bovine submaxillary glands (BSM) type I-S glycoprotein as system suitability and process control, and SW480, SW620 and LS174T human colorectal cancer cell lines and PaTu S and PaTu T human pancreatic cancer cell lines to evaluate the release, recovery and analysis of *O*-glycans. The performance of this approach was evaluated on the basis of a number of criteria such as the ability to detect and characterize *O*-glycans, the repeatability with respect to relative *O*-glycan quantitation and the comparison of the data generated with previously published literature [27, 28].

## Materials and methods

### Materials

The 1.5 mL Eppendorf® Safe-Lock microcentrifuge tubes, potassium hydroxide (KOH), potassium borohydride (KBH_4_), glacial acetic acid, methanol (MeOH), super DHB matrix (2,5-dihydroxybenzoic acid and 2-hydroxy-5-methoxybenzoic acid; 9:1), mucin from bovine submaxillary glands (BSM) type I-S, iodomethane (ICH_3_) and the Parafilm® M sealing film were obtained from Sigma (Dorset, UK). Human colorectal cancer cell lines SW480, SW620 and LS174T, and human pancreatic cancer cell lines PaTu S and PaTu T were obtained from the Department of Surgery at Leiden University Medical Center (Leiden, The Netherlands). The 96-well release plates (4ti-0125), the PCR plates, the foil pierce seals, the semi-automatic heat sealer (HT121TS), the polypropylene collection plates and the silicone plate lids were purchased from 4titude (Surrey, UK). HT permethylation kit (LT-PERMET-VP96) and the cation exchange cartridges (LC-CEX) were obtained from Ludger Ltd (Oxfordshire, UK). The ultrasonic bath (Bandelin Sonorex Digitec DT103H) was from Schalltec (Frankfurt, Germany). Samples were dried down in a Savant centrifugal evaporator from Thermo (Hampshire, UK). All automated steps in the analytical workflow described were performed using a Hamilton MICROLAB STARlet Liquid Handling Workstation from Hamilton Robotics Inc. (Bonaduz, Switzerland).

### Cell culture

SW480, SW620, LS174T, PaTu S and PaTu T cancer cells were cultured as previously described.[29] Briefly, SW620 and SW480 cells were cultured in Hepes-buffered RPMI 1640 culture medium containing L-glutamine and supplemented with penicillin (5000 IU/ml), streptomycin (5 mg/ml), and 10 % (v/v) fetal calf serum (FCS) and LS174T, PaTu S and PaTu T cells were cultured in DMEM medium, supplemented with 10 % (v/v) FCS and antibiotics. Cells were incubated at 37 °C with 5 % CO2 in humidified air and cell culturing was performed up to a confluence of 80 % under sterile conditions. The cell medium was removed and adherent cells were washed twice with 1x PBS and trypsinized using 1x trypsin/EDTA solution in 1x PBS prior to harvesting. To stop trypsin activity, medium in a ratio of 2:5 (trypsin/EDTA/medium; v/v) was added and cells were pelleted at 300 x g for 5 min. Cells were then re-suspended in 3 ml 1x PBS and counted using the CountessTM Automated Cell Counter (Invitrogen, Paisley, UK) based on tryptan blue staining. Cells were aliquoted to 2.0 × 10^6^ cells per ml (1x PBS), and washed twice with 1 ml 1x PBS for 3 min at 1000 x g. The supernatant was removed and pellets stored at -20 °C.

### Disruption of cellular plasma membranes

Cell pellets (~ 2 × 10^6^ cells per sample) were re-suspended by pipetting action in 100 µL of water with resistivity 18.2 MΩ and homogenized for 90 min in a sonication bath alongside BSM type I-S glycoprotein (50 µg per sample) and pure water (100 µL) as a negative control. A thin layer of Parafilm® M sealing film was applied to each Eppendorf tube prior to their sonication in the water bath in order to prevent contamination. Following this step, each sample was manually transferred into a reaction well on the 96-well release plate.

### Automated reductive β-elimination

The sample preparation for *O*-glycan release was performed, as previously described, by semi-automated reductive β-elimination using a Hamilton MICROLAB STARlet Liquid Handling Workstation with the exception of few steps such as off-deck plate sealing, incubation and centrifugal evaporation [20].

### Automated cation-exchange (CEX) cleanup

Released *O*-glycans were purified by automated cation-exchange (CEX) cleanup using LC-CEX cartridges, collected in a polypropylene collection plate and dried down in a centrifugal evaporator [20].

### Automated cleanup by MeOH evaporation

The polypropylene collection plate containing the dried samples was placed onto the robot deck. A 1 mL aliquot of MeOH was dispensed into each well containing samples and the contents were mixed by pipetting action. Following the MeOH addition and mixing, the polypropylene collection plate was placed in a centrifugal evaporator and the contents were dried down completely [20].

### Automated HT permethylation and liquid-liquid extraction

The released and purified *O*-glycans were permethylated using the LudgerTag™ permethylation microplate kit (LT-PERMET-VP96), purified by liquid-liquid extraction (LLE) and dried down in a centrifugal evaporator as previously described [18, 20]. In order to increase the efficiency of the derivatization and reduce the percentage of partial permethylation, a second cycle of permethylation, followed by purification by liquid-liquid extraction, was performed on the robot. Following the second cycle of permethylation, samples were dried down in a centrifugal evaporator. Dried samples were re-suspended in 100 µL of pure water, transferred to a PCR plate and dried down completely in order to be concentrated prior to MALDI-FT-ICR MS analysis.

### MALDI-FT-ICR MS and MS/MS

The permethylated samples were suspended in 10 µL of 70 % MeOH in water. The super DHB MALDI matrix solution (1.5 µL of 5 mg/mL super DHB with 1 mM NaOH in 50 % ACN) was spotted manually on a MTP Anchor-Chip 384 well MALDI-target plate (Bruker Daltonics, Bremen, Germany) followed by 0.5 µL of sample and allowed to dry.

MALDI-FT-ICR MS measurements were performed on a 15T solariX XR instrument equipped with a CombiSource, a ParaCell and a SmarBeam-II laser system (Bruker Daltonics). *O*-glycan spectra were generated from ten acquired scans and 1 M data points in the *m/z* range 207–5000. 200 laser shots were collected per raster using a 500 Hz laser frequency and the “medium” predefined shot pattern. Collision-induced dissociation (CID) experiments were performed with quadrupole (Q1) mass, isolation window and collision energy optimized for each precursor ion. CID fragment ions were measured in the *m/z* range 153–5000. An external calibration of the system was performed prior to the analysis using Caesium iodide (CsI) clusters.

For data analysis and visualization we used Bruker Daltonics DataAnalysis software version 5.0 and MassyTools [30]. Automated data curation, processing and glycan detection was performed using MassyTools version 1.0.2-alpha, build 180703b. [30] MassyTools settings were as follows: the calibration window (peak detection) 0.4, minimum signal-to-noise ratio for calibrants = 3, minimum number of calibrants throughout entire spectrum = 4, charge carrier used for all analytes = sodium, mass modifiers applied to all analytes = per reduced, extraction width = 0.49, background detection method = min, background detection window = 20 min, minimum fraction of total isotopic distribution used for extraction = 0.95. A total of 5 internal calibrants were used prior to *O*-glycan quantitation.

### Glycan representation

Glycan structures were visualized using GlycoWorkBench, version 2.1 [31]. Structures for glycans are depicted following the Consortium for Functional Glycomics (CFG) notation: *N*-acetylglucosamine (N; blue square), fucose (F; red triangle), *N*-acetylgalactosamine (N; yellow square), galactose (H; yellow circle), *N*-acetylneuraminic acid (S; purple diamond), *N*-glycolylneuraminic acid (Sg; light-blue diamond) [32].

## Results and discussion

**Fig. 1:**
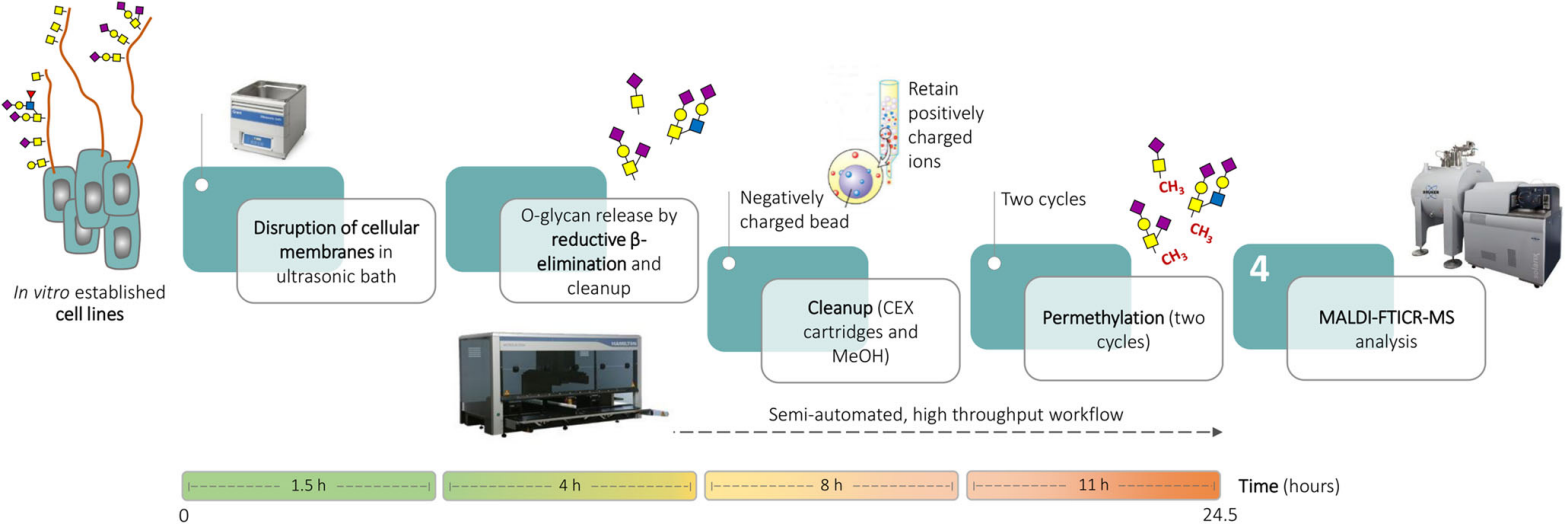
Workflow for semi-automated, high throughput reductive β-elimination, coupled to ultrahigh resolution MALDI-FT-ICR MS for O-glycosylation analysis of in vitro established cell lines

We analysed *O*-glycosylation of human colorectal cancer cell lines SW480, SW620 and LS174T, and human pancreatic cancer cell lines PaTu S and PaTu T using an adaptation of the semi-automated reductive β-elimination method previously described, in combination with MALDI-FT-ICR MS. We took along BSM type I-S glycoprotein as a standard [20, 24, 25]. A few steps of the workflow for semi-automated reductive β-elimination were optimised in order to allow for the liberation of *O*-linked glycans from cancer cell line samples. Given the high complexity of the samples to be analysed, the incubation time in the ultrasonic bath was increased to 4 h in order to allow for a more efficient release of *O*-glycans. Following their release, purification by CEX cleanup and MeOH evaporation, two cycles of permethylation were performed in order to increase the efficiency of the derivatization and reduce the percentage of partial permethylation. A visual representation of the workflow is given in Fig. 1.

The workflow for semi-automated reductive β-elimination release of *O*-glycans was previously validated according to the ICH Q2 (R1) guidelines for the validation of analytical procedures. Therefore, only a partial validation was performed in this study.

The data obtained from the ultrahigh resolution MALDI-FT-ICR mass spectra of all cell lines, BSM type I-S glycoprotein and water blank were extracted and processed using MassyTools [30]. The glycan analytes were automatically included for relative quantitation after triplicate analysis based on the following criteria: average signal-to noise (S/N) of at least 9; average mass error of ± 10 ppm, average isotopic pattern quality (IPQ) score ≤ 0.25. Structural confirmation by MS/MS fragmentation analysis is exemplified for eight glycans (Figs. 6, 7, 8, 9, 10, 11, 12 and 13 in the Online Resource). Proposed *O*-glycan structures were assigned using MS/MS results in combination with information from literature [16].

In order to test the repeatability of the procedure under the same operating conditions, glycans from three independent BSM type I-S samples (50 µg) were released, purified, permethylated twice and analysed by MALDI-FT-ICR MS. Glycan areas extracted after triplicate analysis were integrated and the standard deviations (SDs) and CVs were calculated for the 10 major *O*-glycan structures detected that fitted the selection criteria defined for quantitation. A visual representation of the relative peak areas for the 10 major *O*-glycan structures detected and quantified after triplicate analysis is shown in Fig. 14 of the Online Resource, where error bars represent standard deviation. The CVs generated were ≤ 9.5 % for relative areas (RAs) ≥ 5.2 %. (Table 1 in the Online Resource)

In order to assess intermediate precision or interday variation, the same experimental setup used for the repeatability study was applied, where three BSM type I-S samples (50 µg) were released, purified, permethylated twice and analysed by MALDI-FT-ICR MS on a separate day (day 2). Glycan areas for the 10 major *O*-glycan species highlighted in the previous experiment, extracted after triplicate analysis, were integrated and the CVs were calculated (Table 2 in the Online Resource). The variation between average area values for BSM type I-S *O*-glycans prepared and analyzed on two different days gave CVs ≤ 19.9 % for glycans with relative area ≥ 0.4 %. Higher variation was observed for one *O*-glycan structure (H2N4F2), where the CV peaked at 23.8 % (Table 3 in the Online Resource). We believe that the higher coefficients of variation registered in this experiment are the result of sample preparation variability. This needs to be taken into consideration as a pitfall of the proposed technology.

With regards to the specificity of the method, in order to show that the negative control components do not interfere with released glycans, MALDI-FT-ICR MS spectrum of permethylated *O*-glycans from BSM type I-S was compared with water blank. The water blank sample was analysed in parallel and underwent the same sample processing as all other samples. (Fig. 15 in the Online Resource).

The method was applied for the release and analysis of *O*-glycans from human colorectal cancer cell lines SW480, SW620 and LS174T, and human pancreatic cancer cell lines PaTu S and PaTu T.

**Fig. 2:**
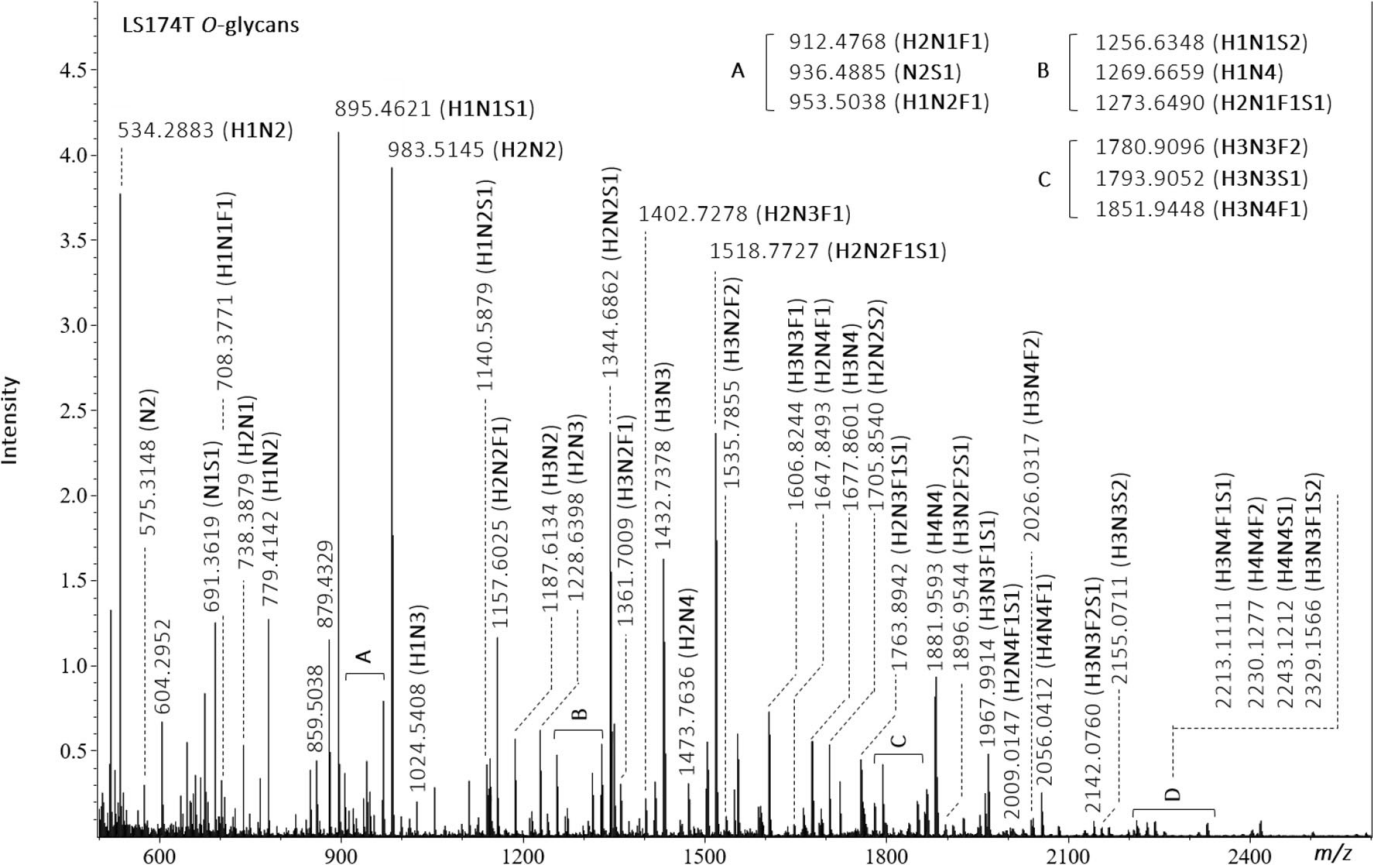
MALDI-FT-ICR MS spectra of LS174T human colorectal cancer cell line with annotated m/z values and compositions for detected O-glycans. Glycan compositions are given in the terms of hexose (H), N-acetylhexosamine (N), deoxyhexose (F), N-acetylneuraminic acid (S), N-glycolylneuraminic acid (Sg)

An example of MALDI-FT-ICR MS spectra obtained for LS174T human colorectal cancer cell line is shown in Fig. 2.

See the Online Resource (Figs. 16, 17, 18 and 19 in the Online Resource) for details regarding the MS spectra generated for SW480, SW620, PaTu S and PaTu T human cancer cell lines. From the analysis of human colorectal cancer cell lines, we were able to detect a total of 9 *O*-glycans in SW480 (Fig. 16 in the Online Resource), 20 *O*-glycans in SW620 (Fig. 17 in the Online Resource) and 46 *O*-glycan structures in LS174T (Fig. 2). With regards to pancreatic cancer cell lines, a total of 39 *O*-glycans were detected in PaTu S (Fig. 18 in the Online Resource), and 12 *O*-glycan structures were detected in PaTu T (Fig. 19 in the Online Resource). For many of the *O*-glycan compositions listed, multiple glycan isomers are possible.

We compared the results obtained from the *O*-glycosylation analysis of the colorectal cancer cell lines evaluated in this study to previously published literature. Although it is known that differences in glycosylation patterns may be observed in cell lines originating from different culture batches, we noticed that the majority of the *O*-glycan structures detected in each cancer cell line had been previously reported. (Tables 4 and 5 in the Online Resource) [16]. From a compositional comparison with previous reports, 4 out of the 5 previously reported *O*-glycans were detected by us for the SW480 cancer cell line [16]. With regards to the SW620 cancer cell line, all 7 previously reported *O*-glycans were reported in this study (Table 4 in the Online Resource). 46 *O*-glycans were detected in this study for LS174T cancer cell line. 13 out of the 16 *O*-glycans reported in the literature for LS174T cancer cell line were also detected with our method (Table 5 in the Online Resource). Of note, 2 out of the 3 *O*-glycans that we were not able to detect, are reported as sulphated species. This may represent a drawback of the technology and will need to be carefully taken into consideration as it is known that sulphated glycans can serve as potential cancer biomarkers [33]. A potential cause for the absence of sulphated *O*-glycan species in this study can be linked to the use of the liquid-liquid extraction process following derivatization by permethylation. In this case, the hydrophilic sulphated glycan species may be lost during phase partitioning.

With regards to PaTu T and PaTu S cancer cell lines we compared the results to a recent publication [34]. 5 out of 12 *O*-glycans detected in this study in PaTu T cell line were also found before [34], while *O*-glycan composition N2H2F1S1 was not detected by our method (Table 6 in the Online Resource). 15 out of 39 *O*-glycans detected in this study in PaTu S cancer cell line were also reported before [34]. Although our method failed to detect *O*-glycans H1N1, H1N3F1S1 and H2N3F2S1, 24 additional *O*-glycan species were reported in this study (Table 7 in the Online Resource). On the other hand, the PGC Nano-LC-ESI-MS/MS technology used for the analysis of *O*-glycans in the previous report [34] can provide advantages such as the detection of *O*-glycan isomers. However, chromatographic separation can hamper detection of low abundant isomers. This data shows that there is no universal approach for the identification of *O*-glycan structures and a combination of methods often needs to be used for a complete characterization of such complex samples.

Glycan areas from triplicate sample analysis of each cell line were integrated and SDs and CVs were calculated for all released *O*-glycans that were included for relative quantitation. The data showing average mass error values (PPM), S/N ratios, IPQ scores, peak areas, SDs and CVs observed for *O*-glycans released from each cell line (Tables 8, 9, 10, 11 and 12 in the Online Resource) provides evidence of a good repeatability.

CVs generated for *O*-glycans released from the colorectal cancer cell line SW480 sporadically peaked at 39.7 %, for *O*-glycan composition H2N2S1 (RA = 14.4 %). 89 % of the *O*-glycan structures detected produced CV ≤ 18.0 for RAs ≥ 0.5 %. (Table 8 in the Online Resource).

All *O*-glycan assignments were within a mass measurement error of ± 4 ppm in a mass range of *m/z* 600 to 1500.

CVs generated for *O*-glycans released from the colorectal cancer cell line SW620 were ≤ 11.6 % for more than 65 % of the analytes detected. The remaining 35 % of the quantified *O*-glycans showed CVs ranging from 12.8 to 24.1 %. (Table 9 in the Online Resource).

Of all *O*-glycan assignments 60 % were within a mass measurement error of ± 3 ppm, with the remaining 40 % within ± 6 ppm, in a mass range of *m/z* 500 to 1800.

For the majority of the *O*-glycans released from the colorectal cancer cell line LS174T, a high degree of reproducibility within the results was observed. 95 % of the *O*-glycan species detected produced CVs ≤ 20 % for RAs ≥ 0.2 %. (Table 10 in the Online Resource). However, a few *O*-glycan peaks showed higher CVs which could be attributed to their low RAs, (24.7 % for glycan composition N2 with RAs of 0.17 %, and 24.9 % for glycan composition H2N4F1S1 with RAs of 0.4 %). We consider that the lower CVs observed for this cancer cell line are the direct result of the higher signal intensities registered on MALDI-FT-ICR MS.

Of all O-glycan assignments 52 % were within a mass measurement error of ± 3 ppm, 15 % within ± 5 ppm, and the remaining 33 % within ± 10 ppm in a mass range of *m/z* 500 to 2400.

In a similar situation to above, for the *O*-glycans released from the pancreatic cancer cell line PaTu S, more than 87 % of the glycan species detected produced CVs ≤ 20 % for RAs ≥ 0.4 %. (Table 11 in the Online Resource). The CVs calculated for lower abundant *O*-glycans with RAs ≤ 2.4 % peaked at 22.9 %, 21.0 %, 34.6 %, 29.9 %, and 23.7 %.

Of all O-glycan assignments 92 % were within a mass measurement error of ± 3 ppm, and 8 % within ± 5 ppm in a mass range of *m/z* 500 to 2400.

Results obtained from the pancreatic cancer cell line PaTu T released *O*-glycans showed less reproducibility than those generated from the other cell lines analysed in this study. Calculated CVs were ≤ 27.7 % for glycans with RAs ≥ 0.5 %. (Table 12 in the Online Resource). This could be explained by the difficulties we faced when re-suspending this cell line in order to obtain a single cell suspension. Given that each cell line presents specific properties with regards to cell to cell adhesion, we believe that there is room for further optimisation in order to promote cell dissociation, with the aim of reducing variation within technical replicates. The detected N1 species produced the highest CV of 55.4 % for an RA of 0.2 %.

Of all O-glycan assignments 75 % were within a mass measurement error of ± 3 ppm, with the remaining 15 % within ± 5 ppm in a mass range of *m/z* 300 to 1800.

**Fig. 3:**
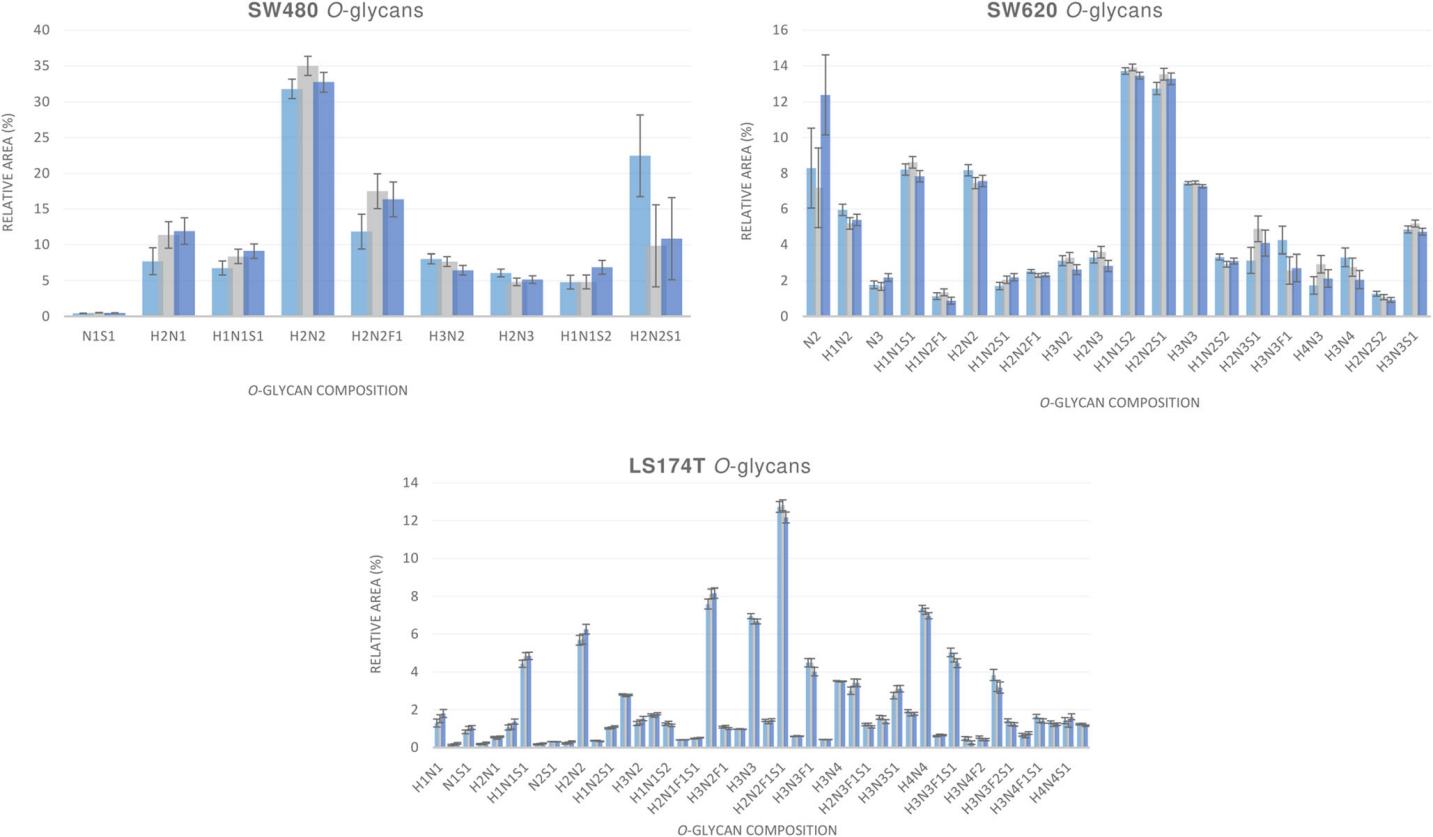
Graphical representation of the relative peak areas for detected O-glycan structures in SW480, SW620 and LS174T human colorectal cancer cell lines. Each bar represents an independent analysis of the same sample aliquot, where the initial sample pellet was split into three technical replicates prior to being processed for O-glycan release, permethylation and MALDI-FT-ICR-MS analysis. Error bars represent the standard deviation calculated per three technical replicates. Glycan compositions are given in the terms of hexose (H), N-acetylhexosamine (N), deoxyhexose (F), N-acetylneuraminic acid (S)

**Fig. 4:**
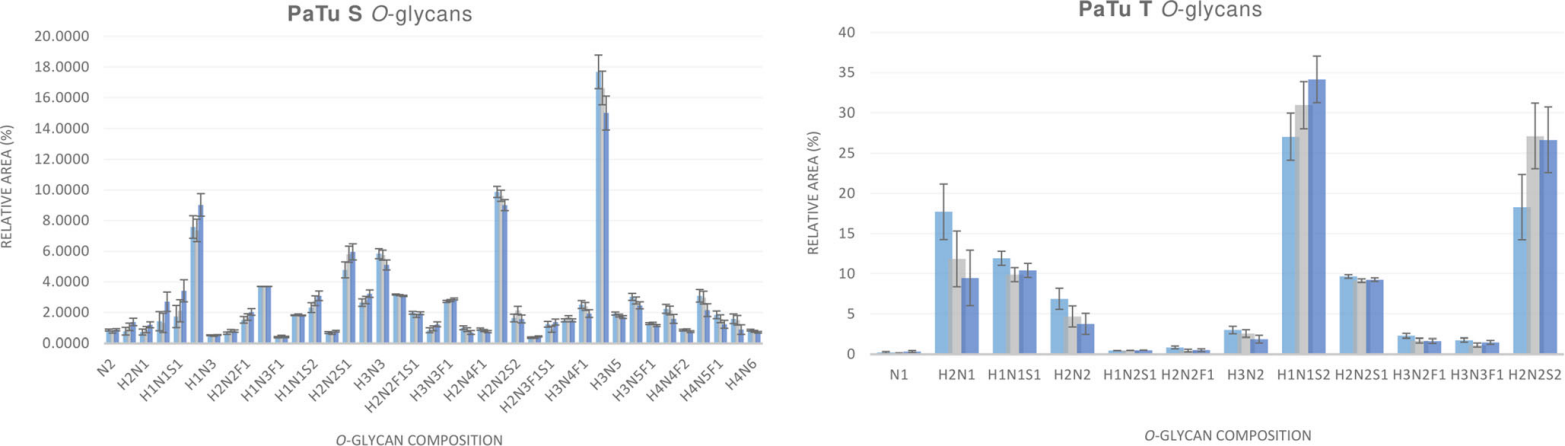
Graphical representation of the relative peak areas for detected O-glycan structures in PaTu S and PaTu T human pancreatic cancer cell lines. Each bar represents an independent analysis of the same sample aliquot, where the initial sample pellet was split into three technical replicates prior to being processed for O-glycan release, permethylation and MALDI-FT-ICR-MS analysis. Error bars represent the standard deviation calculated per three technical replicates. Glycan compositions are given in the terms of hexose (H), N-acetylhexosamine (N), deoxyhexose (F), N-acetylneuraminic acid (S)

A visual representation of the relative peak areas for detected *O*-glycan structures in all cell lines analyzed after triplicate analysis is given in Figs. 3 and 4.

**Fig. 5:**
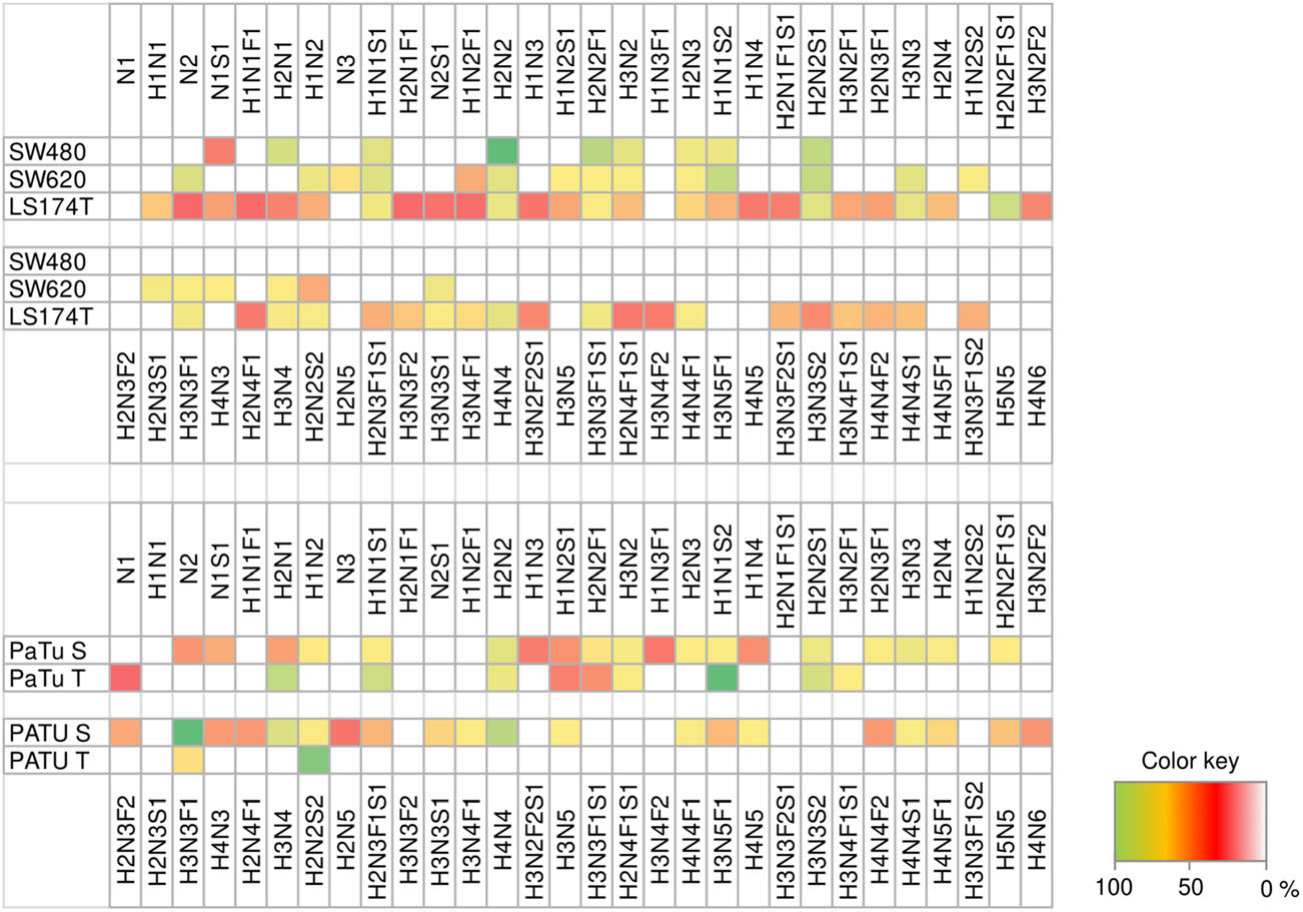
Heat map representing O-glycan distribution in all human cancer cell lines analysed. SW480, SW620 and LS174T human colorectal cancer cell lines are clustered in the top panel. PaTu S and PaTu T human pancreatic cancer cell lines are clustered in the bottom panel. Glycan compositions are given in the terms of hexose (H), N-acetylhexosamine (N), deoxyhexose (F), N-acetylneuraminic acid (S), N-glycolylneuraminic acid (Sg)

A heat map representing *O*-glycan distribution in all human cancer cell lines analysed is shown in Fig. 5, where SW480, SW620 and LS174T human colorectal cancer cell lines are clustered in the top panel while PaTu S, and PaTu T human pancreatic cancer cell lines are clustered in the bottom panel.

We observed additional signals, which are due to permethylation artifacts, that appeared to be in line with O-glycan compositions containing N-glycolylneuraminic acid. These artifact signals produced a series of ions indicating a species that is 30 Da larger than the fully methylated carbohydrate molecules that were observed in PaTu S and LS174T cancer cell lines. These species are believed to be caused by a side reaction involving the permethylation reagents resulting in the incorporation of a methoxymethyl derivative instead of a methyl group. This is a common drawback in the procedure as there are a few permethylation artifacts that may be mis-interpreted as evidence of N-glycolylneuraminic acid-containing glycan species [35, 36].

The optimised, semi-automated, high-throughput reductive β-elimination followed by ultrahigh resolution MALDI-FT-ICR MS analysis has potential for rapid *O*-glycosylation analysis of complex samples such as in-vitro established cell lines. The ultrahigh resolution MALDI-FT-ICR MS allows for detailed glycan identification in complex samples with low mass error measurements. We were able to detect a broad variety of *O*-glycan species, whose compositions ranged from the single to multiple monosaccharides. In order to better appreciate the sub-ppm mass measurement precision of the technology, expanded views of the low mass region of all primary spectra for released O-glycans from colorectal cancer cell lines SW480, SW620 and LS174T, and pancreatic cancer cell lines PaTu S and PaTu T can be observed in Figs. 20, 21, 22, 23 and 24 of the Online Resource. Two MS/MS fragmentation spectra of low molecular mass *O*-glycans are shown in the Online Resource in order to prove that this approach can enable for structural identification of analytes occurring in the matrix-ion region of the spectra (Figs. 25 and 26 in the Online Resource). Of important note is the ability to precisely detect the single permethylated N-acetylhexosaminitol, likely derived from a single GalNAc mucin-type *O*-glycan (Tn antigen). Overall, we believe that this approach represents a valid option for the detection of changes in *O*-glycosylation patterns of biological samples.

We must point out that discrepancies may be observed when comparing the data generated from the newly developed workflow to those previously reported in the literature in relation to the different relative abundance ratios observed for few *O*-glycan species. Specifically, previously published data suggest that the sialylated disaccharides N1S1 and N1Sg1 dominate BSM *O*-glycans, whereas in this study the sialylated trisaccharides N2S1 and N2Sg1 appear to be present in higher relative abundance [27, 28]. However, differences in the quantitation of these oligosaccharides may come from a combination of the different starting material amounts of glycoprotein used, different reagent concentrations and incubation conditions and the use of different analytical methods, such as separation techniques and in particular detection methods.

Furthermore, the stepwise degradation of these oligosaccharides starting at the reducing end and removing one sugar residue at a time (peeling) can hinder the accurate quantitation of the *O*-glycan species detected [37–39]. As previously reported under hydrazinolysis conditions, peeling can be promoted by buffer salts such as divalent cations and other low-molecular-weight materials from glycoprotein samples [37, 39]. Similar matrix effects on peeling may apply under reductive β-elimination conditions, yet further studies are needed to address this. Hence, designing and implementing strategies for containing peeling may help to further improve *O*-glycan analysis with reductive β-elimination [38].

Due to the high throughput potential of this approach, the release, identification and quantitation of *O*-glycans can be performed in a short period of time generating consistent, reliable and reproducible results.

This approach can be used for the *O*-glycosylation analysis of large numbers of biological samples, such as patient sample cohorts, minimizing manual labor and being able to produce accurate and repeatable results.

## Supplementary Information

ESM 1 (DOCX 2004 kb)

ESM 2 (XLSX 173 kb)
