# Supplementary material for: A semi-automated, high throughput approach for O-glycosylation profiling of in vitro established cancer cell lines by MALDI-FT-ICR MS
Source: Glycoconj J. 2021 Jul 20;38(6):747–56. doi: 10.1007/s10719-021-10003-1 (PMC8821499; doi:10.1007/s10719-021-10003-1)
Supplement: Supplementary file 1 — (DOCX 2004 kb) [file 10719_2021_10003_MOESM1_ESM.docx]

A semi-automated, high throughput approach for *O*-glycosylation profiling of in vitro established cancer cell lines by MALDI-FT-ICR-MS

Maximilianos Kotsias^1*^, Katarina Madunic^2^, Simone Nicolardi^2^, Radoslaw P. Kozak^1¶^, Richard A. Gardner^1¶^, Bas C. Jansen^1^, Daniel I. R. Spencer^1^, Manfred Wuhrer^2^

^1^Ludger Ltd., Culham Science Centre, Abingdon, Oxfordshire, UK.

^2^Leiden University Medical Centre, Centre for Proteomics and Metabolomics, Leiden, The Netherlands.

^¶^These authors contributed equally to this work.

***Corresponding author**

E-mail: maximilianos.kotsias@ludger.com

Supporting Information


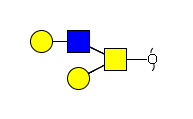

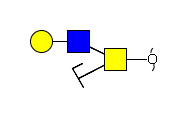

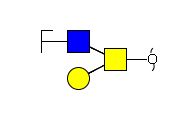

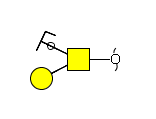

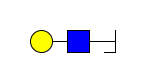


400

450

500

550

600

650

700

750

800

850

900

950

1000

30

60

90

747.3863

486.2303

520.2699

Intensity (%)

*m/z*

m/z 983.5145, [M + Na]^+^ (H2N2)

690.3288

806.5051

833.2787

659.2855

643.2902

**Fig. 6.** Fragment ion spectrum of precursor *m/z* 983.5145, [M + Na]^+^ from SW480 human colorectal cancer cell line *O*-glycans.


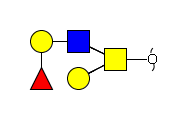

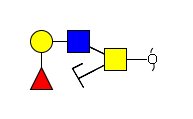

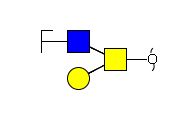

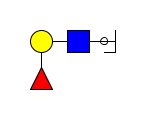

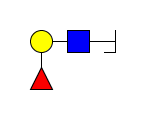

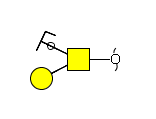

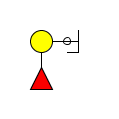


400

500

600

700

800

900

1000

1100

30

60

90

Intensity (%)

*m/z*

m/z 1157.6020, [M + Na]^+^ (H2N2F1)

433.2049

520.2731

660.3201

678.3303

747.3882

921.4784

678.3303

690.3315

983.5144

**Fig. 7.** Fragment ion spectrum of precursor *m/z* 1157.6020, [M + Na]^+^ from SW480 human colorectal cancer cell line *O*-glycans. Various glycan isomers may be present.


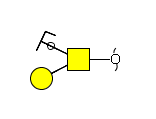

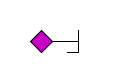

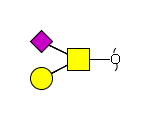


250

300

350

400

450

500

550

600

650

700

750

800

850

900

30

60

90

Intensity (%)

*m/z*

m/z 895.4612, [M + Na]^+^ (H1N1S1)

398.1748

520.2719


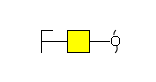


298.1627


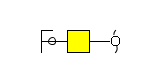


316.1705


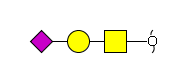


258.2794

430.9635

452.8453

620.2881

544.9721

**Fig. 8.** Fragment ion spectrum of precursor *m/z* 895.4612, [M + Na]^+^ from SW620 human colorectal cancer cell line *O*-glycans. Various glycan isomers may be present.


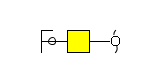

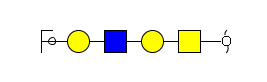

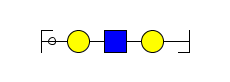

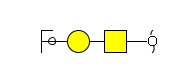

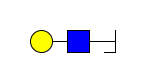

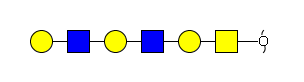


300

600

900

1200

30

60

90

Intensity (%)

*m/z*

m/z 1432.7360, [M + Na]^+^ (H3N3)

316.1727

486.2313

520.2731


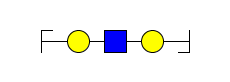


658.3023

676.3104


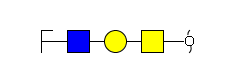


747.3886


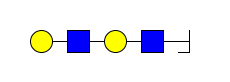


935.4546

969.4983

477.5843

1196.6105

857.2777

**Fig. 9.** Fragment ion spectrum of precursor *m/z* 1432.7360, [M + Na]^+^ from SW620 human colorectal cancer cell line *O*-glycans. Various glycan isomers may be present.


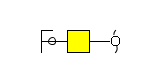

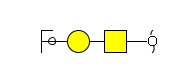


300

600

900

1200

5

10

15

20

25

30

35

Intensity (%)

*m/z*

m/z 1344.6882. [M + Na]^+^ (H2N2S1)


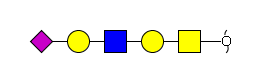


316.1725


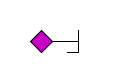


398.1769


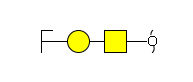


502.2604

520.2731


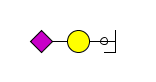


620.2891


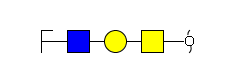


747.3882


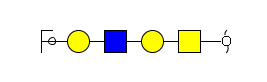


969.4981

362.9724

398.1769

486.2317

580.7558

610.9450

**Fig. 10.** Fragment ion spectrum of precursor *m/z* 1344.6882, [M + Na]^+^ from PaTu T human pancreatic cancer cell line *O*-glycans. Various glycan isomers may be present.


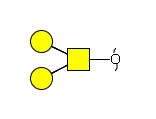

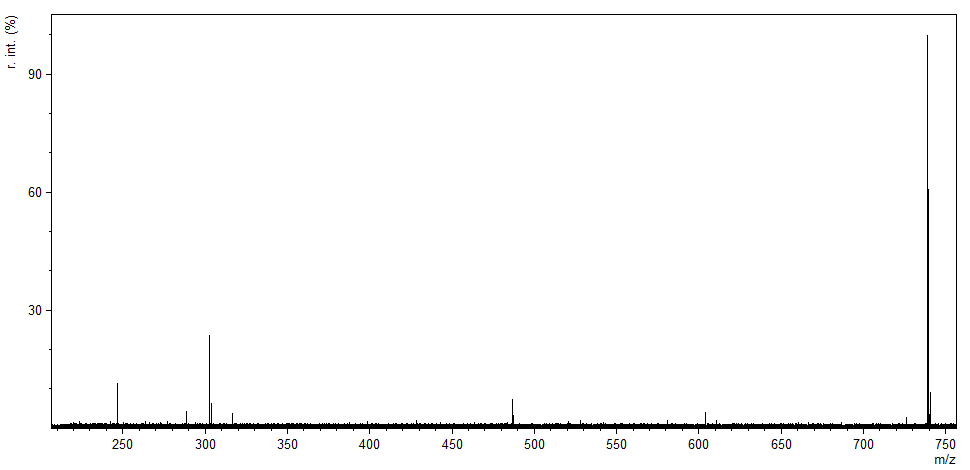


Intensity (%)

m/z 738.3871. [M + Na]^+^ (H2N1)


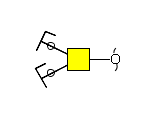


302.3527

246.1605

486.2242

**Fig. 11.** Fragment ion spectrum of precursor *m/z* 738.3871, [M + Na]^+^ from PaTu T human pancreatic cancer cell line *O*-glycans. Various glycan isomers may be present.


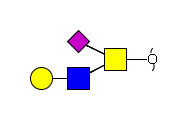

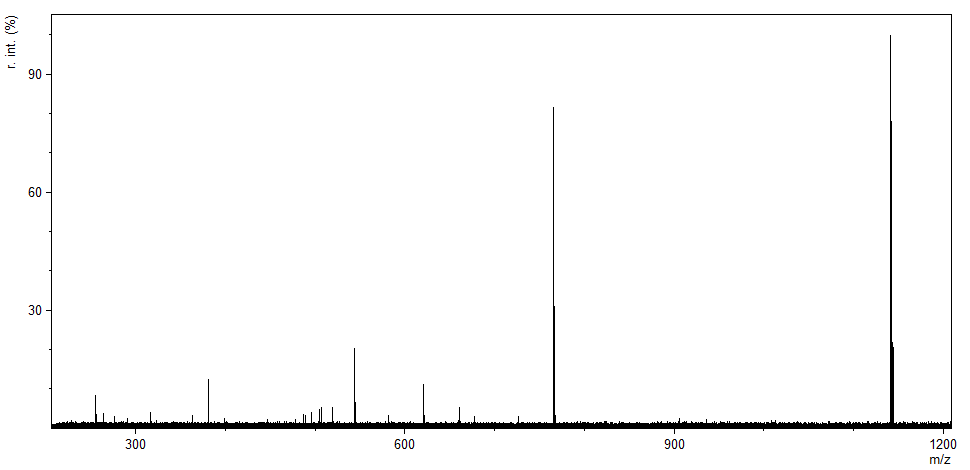


Intensity (%)

m/z 1140.5885. [M + Na]^+^ (H1N2S1)


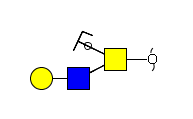


765.2206


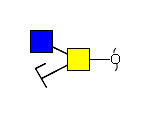


543.2407


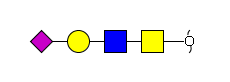

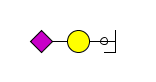


620.4775

380.2526

**Fig. 12.** Fragment ion spectrum of precursor *m/z* 1140.5885, [M + Na]^+^ from SW620 human colorectal cancer cell line *O*-glycans. Various glycan isomers may be present.


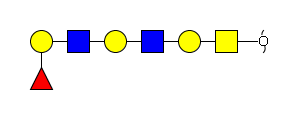

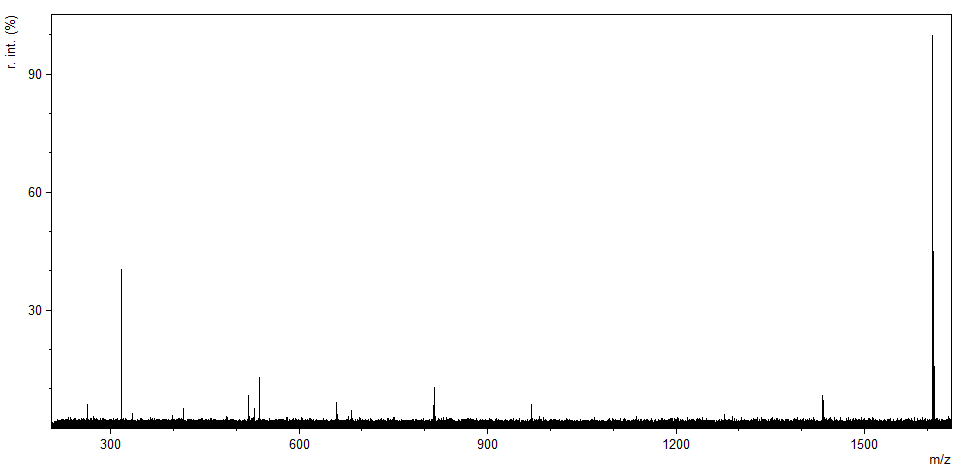

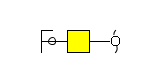


316.3615


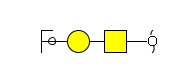


520.1078

535.2330

814.6056


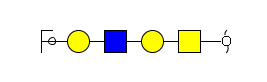


969.4172

1432.9622

Intensity (%)

m/z 1606.8299. [M + Na]^+^ (H3N3F1)

**Fig. 13.** Fragment ion spectrum of precursor *m/z* 1606.8299, [M + Na]^+^ from PaTu S human pancreatic cancer cell line *O*-glycans. Various glycan isomers may be present.

**Fig. 14.** Graphical representation of the relative peak areas for detected *O*-glycan structures in BSM type I-S glycoprotein calculated after triplicate analysis of technical replicates. Error bars represent standard deviation. Glycan compositions are given in the terms of hexose (H), *N*-acetylhexosamine (N), deoxyhexose (F), *N*-acetylneuraminic acid (S), *N*-glycolylneuraminic acid (Sg)

**Table 1.** Repeatability of the procedure (day 1). Glycan compositions, calculated *m/z* values, average registered *m/z* values from three sample replicates (Avg. *m/z*), relative areas, mass precision (PPM), index of peak quality (IPQ), signal-to-noise (S/N) ratios, standard deviations (SDs) and coefficients of variation (CVs) for 10 major *O*-glycan structures detected in BSM type I-S glycoprotein and calculated after triplicate analysis. Glycan compositions are given in the terms of hexose (H), *N*-acetylhexosamine (N), deoxyhexose (F), *N*-acetylneuraminic acid (S), *N*-glycolylneuraminic acid (Sg)

| Peak ID | Composition | | | | | *m/z*  (calculated) | Avg. *m/z*  (registered) | Criteria | BSM type I-S *O*-glycan data (Day 1) | | | | Variation | |
| --- | --- | --- | --- | --- | --- | --- | --- | --- | --- | --- | --- | --- | --- | --- |
|  |  |  |  |  |  |  |  |  | Replicate A | Replicate B | Replicate C | Average | SD | **CV** |
|  | Hex (H) | HexNAc (N) | Fuc (F) | Neu5Ac (S) | Neu5Gc (Sg) |  |  |  |  |  |  |  |  |  |
| A | 0 | 2 | 0 | 1 | 0 | 936.4887 | 936.4882 | Relative % area | 12.71 | 15.59 | 15.72 | 14.68 | 1.39 | **9.46** |
|  |  |  |  |  |  |  |  | Mass precision (PPM) | 0.36 | 0.39 | 0.71 | 0.49 |  |  |
|  |  |  |  |  |  |  |  | Index of peak quality (IPQ) | 0.04 | 0.05 | 0.05 | 0.05 |  |  |
|  |  |  |  |  |  |  |  | Signal-to-noise ratio (S/N) | 5743.67 | 5893.81 | 7157.13 | 6264.87 |  |  |
| B | 0 | 2 | 0 | 0 | 1 | 966.4993 | 966.4982 | Relative % area | 11.46 | 12.51 | 13.56 | 12.51 | 0.86 | **6.84** |
|  |  |  |  |  |  |  |  | Mass precision (PPM) | 0.95 | 0.93 | 1.44 | 1.11 |  |  |
|  |  |  |  |  |  |  |  | Index of peak quality (IPQ) | 0.06 | 0.06 | 0.06 | 0.06 |  |  |
|  |  |  |  |  |  |  |  | Signal-to-noise ratio (S/N) | 7490.03 | 5049.16 | 3887.93 | 5475.71 |  |  |
| C | 1 | 3 | 1 | 0 | 0 | 1198.6303 | 1198.6297 | Relative % area | 4.83 | 5.85 | 4.80 | 5.16 | 0.49 | **9.48** |
|  |  |  |  |  |  |  |  | Mass precision (PPM) | 0.51 | 0.55 | 0.35 | 0.47 |  |  |
|  |  |  |  |  |  |  |  | Index of peak quality (IPQ) | 0.02 | 0.02 | 0.02 | 0.02 |  |  |
|  |  |  |  |  |  |  |  | Signal-to-noise ratio (S/N) | 1980.02 | 2144.53 | 1617.29 | 1913.95 |  |  |
| D | 1 | 2 | 1 | 1 | 0 | 1314.6777 | 1314.6759 | Relative % area | 10.61 | 10.74 | 10.30 | 10.55 | 0.19 | **1.76** |
|  |  |  |  |  |  |  |  | Mass precision (PPM) | 1.21 | 1.29 | 1.59 | 1.36 |  |  |
|  |  |  |  |  |  |  |  | Index of peak quality (IPQ) | 0.06 | 0.06 | 0.06 | 0.06 |  |  |
|  |  |  |  |  |  |  |  | Signal-to-noise ratio (S/N) | 1822.33 | 1613.71 | 1728.35 | 1721.46 |  |  |
| E | 2 | 2 | 2 | 0 | 0 | 1331.6930 | 1331.6913 | Relative % area | 13.66 | 14.27 | 13.35 | 13.76 | 0.38 | **2.79** |
|  |  |  |  |  |  |  |  | Mass precision (PPM) | 1.07 | 1.16 | 1.70 | 1.31 |  |  |
|  |  |  |  |  |  |  |  | Index of peak quality (IPQ) | 0.02 | 0.02 | 0.02 | 0.02 |  |  |
|  |  |  |  |  |  |  |  | Signal-to-noise ratio (S/N) | 1930.39 | 2713.30 | 2688.78 | 2444.16 |  |  |
| F | 2 | 2 | 0 | 1 | 0 | 1344.6882 | 1344.6878 | Relative % area | 6.48 | 5.93 | 6.27 | 6.23 | 0.23 | **3.64** |
|  |  |  |  |  |  |  |  | Mass precision (PPM) | 0.18 | 0.27 | 0.38 | 0.28 |  |  |
|  |  |  |  |  |  |  |  | Index of peak quality (IPQ) | 0.06 | 0.08 | 0.08 | 0.07 |  |  |
|  |  |  |  |  |  |  |  | Signal-to-noise ratio (S/N) | 1415.60 | 749.46 | 843.20 | 1002.75 |  |  |
| G | 1 | 4 | 1 | 0 | 0 | 1443.7576 | 1443.7560 | Relative % area | 5.34 | 5.15 | 5.19 | 5.23 | 0.08 | **1.59** |
|  |  |  |  |  |  |  |  | Mass precision (PPM) | 0.96 | 1.08 | 1.23 | 1.09 |  |  |
|  |  |  |  |  |  |  |  | Index of peak quality (IPQ) | 0.03 | 0.05 | 0.03 | 0.04 |  |  |
|  |  |  |  |  |  |  |  | Signal-to-noise ratio (S/N) | 638.52 | 901.57 | 751.29 | 763.79 |  |  |
| H | 1 | 3 | 1 | 1 | 0 | 1559.8040 | 1559.8037 | Relative % area | 9.97 | 8.39 | 8.90 | 9.08 | 0.66 | **7.24** |
|  |  |  |  |  |  |  |  | Mass precision (PPM) | 0.01 | 0.16 | 0.44 | 0.20 |  |  |
|  |  |  |  |  |  |  |  | Index of peak quality (IPQ) | 0.06 | 0.06 | 0.07 | 0.07 |  |  |
|  |  |  |  |  |  |  |  | Signal-to-noise ratio (S/N) | 601.24 | 792.43 | 790.89 | 728.19 |  |  |
| K | 2 | 3 | 2 | 0 | 0 | 1576.8193 | 1576.8184 | Relative % area | 14.34 | 12.66 | 12.85 | 13.28 | 0.75 | **5.66** |
|  |  |  |  |  |  |  |  | Mass precision (PPM) | 0.64 | 0.47 | 0.68 | 0.59 |  |  |
|  |  |  |  |  |  |  |  | Index of peak quality (IPQ) | 0.04 | 0.04 | 0.03 | 0.04 |  |  |
|  |  |  |  |  |  |  |  | Signal-to-noise ratio (S/N) | 1433.44 | 2471.49 | 2114.60 | 2006.51 |  |  |
| L | 2 | 4 | 2 | 0 | 0 | 1821.9456 | 1821.9446 | Relative % area | 10.60 | 8.91 | 9.06 | 9.53 | 0.76 | **8.00** |
|  |  |  |  |  |  |  |  | Mass precision (PPM) | 0.44 | 0.36 | 0.89 | 0.56 |  |  |
|  |  |  |  |  |  |  |  | Index of peak quality (IPQ) | 0.04 | 0.05 | 0.05 | 0.05 |  |  |
|  |  |  |  |  |  |  |  | Signal-to-noise ratio (S/N) | 953.74 | 1827.17 | 1767.57 | 1516.16 |  |  |

**Table 2.** Repeatability of the procedure (day 2). Glycan compositions, calculated *m/z* values, average registered *m/z* values from three sample replicates (Avg. *m/z*), relative areas, mass precision (PPM), index of peak quality (IPQ), signal-to-noise (S/N) ratios, standard deviations (SDs) and coefficients of variation (CVs) for 10 major *O*-glycan structures detected in BSM type I-S glycoprotein and calculated after triplicate analysis. Triplicate of BSM type I-S samples were released, permethylated and analysed on day 2 in order to assess intermediate precision (interday variation) as a comparison to the BSM type I-S samples released, permethylated and analysed by MALDI-FTICR-MS on day 1. Glycan compositions are given in the terms of hexose (H), *N*-acetylhexosamine (N), deoxyhexose (F), *N*-acetylneuraminic acid (S), *N*-glycolylneuraminic acid (Sg)

| Peak ID | Composition | | | | | *m/z*  (calculated) | Avg. *m/z*  (registered) | Criteria | BSM type I-S *O*-glycan data (Day 2) | | | | Variation | |
| --- | --- | --- | --- | --- | --- | --- | --- | --- | --- | --- | --- | --- | --- | --- |
|  |  |  |  |  |  |  |  |  | Replicate A | Replicate B | Replicate C | Average | SD | **CV** |
|  | Hex (H) | HexNAc (N) | Fuc (F) | Neu5Ac (S) | Neu5Gc (Sg) |  |  |  |  |  |  |  |  |  |
| A | 0 | 2 | 0 | 1 | 0 | 936.4887 | 936.4883 | Relative % area | 22.35 | 18.42 | 19.66 | 20.14 | 1.64 | **8.14** |
|  |  |  |  |  |  |  |  | Mass precision (PPM) | 0.05 | 0.24 | 0.89 | 0.40 |  |  |
|  |  |  |  |  |  |  |  | Index of peak quality (IPQ) | 0.05 | 0.05 | 0.04 | 0.05 |  |  |
|  |  |  |  |  |  |  |  | Signal-to-noise ratio (S/N) | 3489.89 | 5085.74 | 7891.51 | 5489.04 |  |  |
| B | 0 | 2 | 0 | 0 | 1 | 966.4993 | 966.4984 | Relative % area | 16.64 | 13.62 | 14.12 | 14.79 | 1.32 | **8.92** |
|  |  |  |  |  |  |  |  | Mass precision (PPM) | 0.92 | 1.06 | 0.92 | 0.96 |  |  |
|  |  |  |  |  |  |  |  | Index of peak quality (IPQ) | 0.06 | 0.06 | 0.06 | 0.06 |  |  |
|  |  |  |  |  |  |  |  | Signal-to-noise ratio (S/N) | 5082.55 | 5719.92 | 10262.18 | 7021.55 |  |  |
| C | 1 | 3 | 1 | 0 | 0 | 1198.6303 | 1198.6296 | Relative % area | 5.02 | 5.50 | 5.08 | 5.20 | 0.21 | **4.08** |
|  |  |  |  |  |  |  |  | Mass precision (PPM) | 0.69 | 0.54 | 0.49 | 0.57 |  |  |
|  |  |  |  |  |  |  |  | Index of peak quality (IPQ) | 0.03 | 0.02 | 0.02 | 0.03 |  |  |
|  |  |  |  |  |  |  |  | Signal-to-noise ratio (S/N) | 1539.44 | 2393.78 | 1086.20 | 1673.14 |  |  |
| D | 1 | 2 | 1 | 1 | 0 | 1314.6777 | 1314.6762 | Relative % area | 12.23 | 13.79 | 14.46 | 13.49 | 0.93 | **6.92** |
|  |  |  |  |  |  |  |  | Mass precision (PPM) | 1.01 | 0.91 | 1.40 | 1.11 |  |  |
|  |  |  |  |  |  |  |  | Index of peak quality (IPQ) | 0.11 | 0.11 | 0.08 | 0.10 |  |  |
|  |  |  |  |  |  |  |  | Signal-to-noise ratio (S/N) | 1490.02 | 1670.29 | 2730.49 | 1963.60 |  |  |
|  |  |  |  |  |  |  |  |  |  |  |  |  |  |  |
|  |  |  |  |  |  |  |  |  |  |  |  |  |  |  |
| E | 2 | 2 | 2 | 0 | 0 | 1331.6930 | 1331.6916 | Relative % area | 11.47 | 14.23 | 12.85 | 12.85 | 1.13 | **8.76** |
|  |  |  |  |  |  |  |  | Mass precision (PPM) | 0.87 | 0.77 | 1.41 | 1.02 |  |  |
|  |  |  |  |  |  |  |  | Index of peak quality (IPQ) | 0.02 | 0.02 | 0.02 | 0.02 |  |  |
|  |  |  |  |  |  |  |  | Signal-to-noise ratio (S/N) | 1908.04 | 1724.37 | 2583.21 | 2071.87 |  |  |
| F | 2 | 2 | 0 | 1 | 0 | 1344.6882 | 1344.6876 | Relative % area | 6.15 | 6.72 | 7.70 | 6.86 | 0.64 | **9.30** |
|  |  |  |  |  |  |  |  | Mass precision (PPM) | 0.48 | 0.39 | 0.51 | 0.46 |  |  |
|  |  |  |  |  |  |  |  | Index of peak quality (IPQ) | 0.04 | 0.06 | 0.05 | 0.05 |  |  |
|  |  |  |  |  |  |  |  | Signal-to-noise ratio (S/N) | 753.75 | 823.24 | 1563.79 | 1046.93 |  |  |
| G | 1 | 4 | 1 | 0 | 0 | 1443.7576 | 1443.7564 | Relative % area | 3.73 | 3.71 | 3.29 | 3.58 | 0.20 | **5.64** |
|  |  |  |  |  |  |  |  | Mass precision (PPM) | 0.73 | 0.68 | 1.09 | 0.83 |  |  |
|  |  |  |  |  |  |  |  | Index of peak quality (IPQ) | 0.03 | 0.07 | 0.03 | 0.04 |  |  |
|  |  |  |  |  |  |  |  | Signal-to-noise ratio (S/N) | 499.42 | 771.28 | 539.94 | 603.55 |  |  |
| H | 1 | 3 | 1 | 1 | 0 | 1559.8040 | 1559.8038 | Relative % area | 7.72 | 8.06 | 8.34 | 8.04 | 0.25 | **3.15** |
|  |  |  |  |  |  |  |  | Mass precision (PPM) | 0.00 | 0.02 | 0.35 | 0.12 |  |  |
|  |  |  |  |  |  |  |  | Index of peak quality (IPQ) | 0.06 | 0.08 | 0.07 | 0.07 |  |  |
|  |  |  |  |  |  |  |  | Signal-to-noise ratio (S/N) | 475.34 | 615.83 | 820.03 | 637.07 |  |  |
| K | 2 | 3 | 2 | 0 | 0 | 1576.8193 | 1576.8178 | Relative % area | 8.67 | 9.93 | 8.55 | 9.05 | 0.63 | **6.91** |
|  |  |  |  |  |  |  |  | Mass precision (PPM) | 0.90 | 0.90 | 1.02 | 0.94 |  |  |
|  |  |  |  |  |  |  |  | Index of peak quality (IPQ) | 0.04 | 0.03 | 0.04 | 0.03 |  |  |
|  |  |  |  |  |  |  |  | Signal-to-noise ratio (S/N) | 1480.94 | 1969.40 | 1787.07 | 1745.80 |  |  |
| L | 2 | 4 | 2 | 0 | 0 | 1821.9456 | 1821.9449 | Relative % area | 6.03 | 6.01 | 5.96 | 6.00 | 0.03 | **0.46** |
|  |  |  |  |  |  |  |  | Mass precision (PPM) | 0.16 | 0.22 | 0.69 | 0.36 |  |  |
|  |  |  |  |  |  |  |  | Index of peak quality (IPQ) | 0.04 | 0.04 | 0.04 | 0.04 |  |  |
|  |  |  |  |  |  |  |  | Signal-to-noise ratio (S/N) | 994.66 | 1629.67 | 1592.03 | 1405.45 |  |  |

**Table 3.** Intermediate precision of the procedure. Glycan compositions, calculated *m/z* values, relative areas, mass precision (PPM), index of peak quality (IPQ), signal-to-noise (S/N) ratios, standard deviations (SDs) and coefficients of variation (CVs) for 10 major *O*-glycan structures detected in BSM type I-S glycoprotein and calculated after triplicate analysis. Glycans from three independent BSM type I-S samples were released, permethylated and analysed by MALDI-FTICR-MS in two separate days (three samples on day 1 versus 3 samples on day 2) to assess intermediate precision (interday variation). Glycan compositions are given in the terms of hexose (H), *N*-acetylhexosamine (N), deoxyhexose (F), *N*-acetylneuraminic acid (S), *N*-glycolylneuraminic acid (Sg).

| Peak ID | Composition | | | | | *m/z*  (calculated) | BSM type I-S *O*-glycan data (Day 1 vs Day 2) | | | | | | | Variation | |
| --- | --- | --- | --- | --- | --- | --- | --- | --- | --- | --- | --- | --- | --- | --- | --- |
|  |  |  |  |  |  |  | Relative % area | | | | | | Average | SD | **CV** |
|  | Hex (H) | HexNAc (N) | Fuc (F) | Neu5Ac (S) | Neu5Gc (Sg) |  | Replicate A | Replicate B | Replicate C | Replicate D | Replicate E | Replicate F |  |  |  |
| A | 0 | 2 | 0 | 1 | 0 | 936.4887 | 12.71 | 15.59 | 15.72 | 22.35 | 18.42 | 19.66 | 17.41 | 3.13 | **17.96** |
| B | 0 | 2 | 0 | 0 | 1 | 966.4993 | 11.46 | 12.51 | 13.56 | 16.64 | 13.62 | 14.12 | 13.65 | 1.59 | **11.68** |
| C | 1 | 3 | 1 | 0 | 0 | 1198.6303 | 4.83 | 5.85 | 4.80 | 5.02 | 5.50 | 5.08 | 5.18 | 0.38 | **7.29** |
| D | 1 | 2 | 1 | 1 | 0 | 1314.6777 | 10.61 | 10.74 | 10.30 | 12.23 | 13.79 | 14.46 | 12.02 | 1.62 | **13.47** |
| E | 2 | 2 | 2 | 0 | 0 | 1331.6930 | 13.66 | 14.27 | 13.35 | 11.47 | 14.23 | 12.85 | 13.31 | 0.96 | **7.19** |
| F | 2 | 2 | 0 | 1 | 0 | 1344.6882 | 6.48 | 5.93 | 6.27 | 6.15 | 6.72 | 7.70 | 6.54 | 0.57 | **8.75** |
| G | 1 | 4 | 1 | 0 | 0 | 1443.7576 | 5.34 | 5.15 | 5.19 | 3.73 | 3.71 | 3.29 | 4.40 | 0.84 | **19.06** |
| H | 1 | 3 | 1 | 1 | 0 | 1559.8040 | 9.97 | 8.39 | 8.90 | 7.72 | 8.06 | 8.34 | 8.56 | 0.72 | **8.43** |
| K | 2 | 3 | 2 | 0 | 0 | 1576.8193 | 14.34 | 12.66 | 12.85 | 8.67 | 9.93 | 8.55 | 11.17 | 2.23 | **19.94** |
| L | 2 | 4 | 2 | 0 | 0 | 1821.9456 | 10.60 | 8.91 | 9.06 | 6.03 | 6.01 | 5.96 | 7.76 | 1.84 | **23.75** |

**Fig. 15.** Specificity of the procedure. MALDI-FTICR-MS of permethylated *O*-glycans from BSM type I-S compared with water blank (negative control) analysed in parallel where the water blank underwent the same sample processing as BSM type I-S sample. Y axis is normalized to show that the negative control components do not interfere with released glycans and demonstrate specificity of the method

**Fig. 16.** MALDI-FTICR-MS spectra of SW480 human colorectal cancer cell line with annotated m/z values and compositions for detected *O*-glycans. Glycan compositions are given in the terms of hexose (H), N-acetylhexosamine (N), deoxyhexose (F), N-acetylneuraminic acid (S).

**Fig. 17.** MALDI-FTICR-MS spectra of SW620 human colorectal cancer cell line with annotated m/z values and compositions for detected *O*-glycans. Glycan compositions are given in the terms of hexose (H), N-acetylhexosamine (N), deoxyhexose (F), N-acetylneuraminic acid (S).


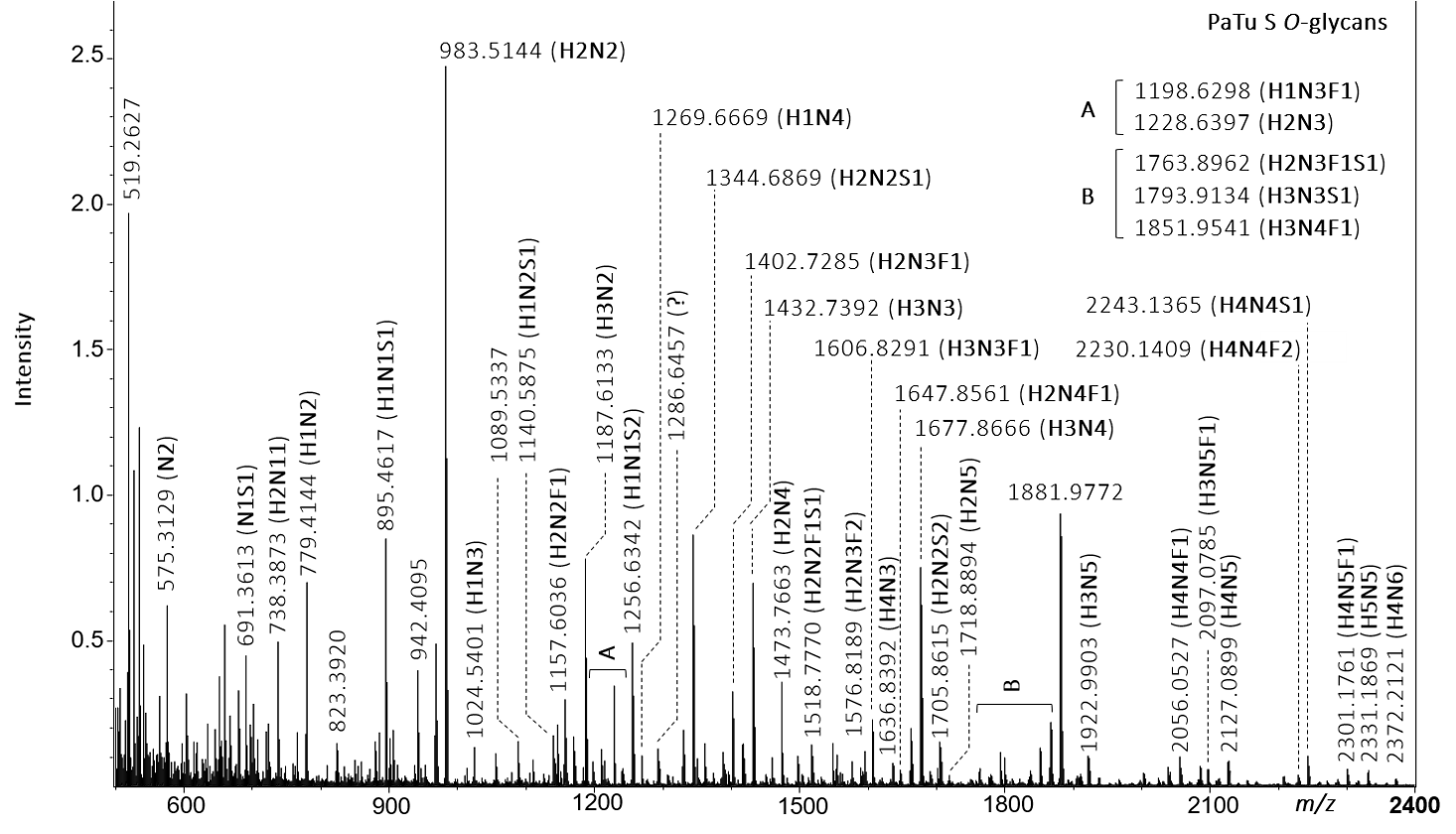


**Fig. 18.** MALDI-FTICR-MS spectra of PaTu S human pancreatic cancer cell line with annotated m/z values and compositions for detected *O*-glycans. Glycan compositions are given in the terms of hexose (H), N-acetylhexosamine (N), deoxyhexose (F), N-acetylneuraminic acid (S).

**Fig. 19.** MALDI-FTICR-MS spectra of PaTu T human pancreatic cancer cell line with annotated m/z values and compositions for detected *O*-glycans. Glycan compositions are given in the terms of hexose (H), N-acetylhexosamine (N), deoxyhexose (F), N-acetylneuraminic acid (S).

**Table 4.** Comparison of the *O*-glycan species identified from the glycosylation analysis of colorectal cancer cell lines SW480 and SW620 with previously reported *O*-glycan species from the literature. *O*-glycan compositions detected in this study and/or previously reported in the literature are highlighted in green. *O*-glycan compositions not detected in this study and/or not reported in the literature are highlighted in red.[1] Glycan compositions are given in the terms of hexose (H), N-acetylhexosamine (N), deoxyhexose (F), N-acetylneuraminic acid (S)**.**

| SW480 *O*-glycans | | | |  | SW620 *O*-glycans | | | |
| --- | --- | --- | --- | --- | --- | --- | --- | --- |
| Peak ID | Composition | Detected (in this work) | Literature[1] (previously reported) |  | Peak ID | Composition | Detected (in this work) | Literature[1] (previously reported |
| A | H1S1 | Yes | No |  | **A** | N2 | Yes | No |
| B | H2N1 | Yes | No |  | **B** | H1N2 | Yes | Yes |
| C | H1N1S1 | Yes | Yes |  | **C** | N3 | Yes | No |
| D | H2N2 | Yes | Yes |  | **D** | H1N1S1 | Yes | Yes |
| E | H2N2F1 | Yes | No |  | **E** | H1N2F1 | Yes | No |
| F | H3N2 | Yes | No |  | **F** | H2N2 | Yes | Yes |
| G | H2N3 | Yes | No |  | **G** | H1N2S1 | Yes | No |
| H | H1N1S2 | Yes | Yes |  | **H** | H2N2F1 | Yes | No |
| I | H2N2S1 | Yes | Yes |  | **I** | H3N2 | Yes | No |
| J | H2N2S2 | No | Yes |  | **J** | H2N3 | Yes | No |
|  |  |  |  |  | **K** | H1N1S2 | Yes | Yes |
|  |  |  |  |  | **L** | H2N2S1 | Yes | Yes |
|  |  |  |  |  | **M** | H3N3 | Yes | Yes |
|  |  |  |  |  | **N** | H1N2S2 | Yes | No |
|  |  |  |  |  | **O** | H2N3S1 | Yes | No |
|  |  |  |  |  | **P** | H3N3F1 | Yes | No |
|  |  |  |  |  | **Q** | H4N3 | Yes | No |
|  |  |  |  |  | **R** | H3N4 | Yes | No |
|  |  |  |  |  | **S** | H2N2S2 | Yes | Yes |
|  |  |  |  |  | **T** | H3N3S1 | Yes | No |

**Table 5.** Comparison of the *O*-glycan species identified from the glycosylation analysis of colorectal cancer cell line LS174T with previously reported *O*-glycan species from the literature. *O*-glycan compositions detected in this study and/or previously reported in the literature are highlighted in green. *O*-glycan compositions not detected in this study and/or not reported in the literature are highlighted in red.[1] Glycan compositions are given in the terms of hexose (H), *N*-acetylhexosamine (N), deoxyhexose (F), *N*-acetylneuraminic acid (S), *N*-glycolylneuraminic acid (Sg), sulphated structure (*su*).

| LS174T *O*-glycans | | | |  | LS174T *O*-glycans | | | |
| --- | --- | --- | --- | --- | --- | --- | --- | --- |
| Peak ID | Composition | Detected (in this work) | Literature[1] (previously reported) |  | Peak ID | Composition | Detected (in this work) | Literature[1] (previously reported |
| A | H1N1 | Yes | No |  | **Y** | H2N2F1S1 | Yes | Yes |
| B | N2 | Yes | No |  | **Z** | H3N2F2 | Yes | No |
| C | N1S1 | Yes | Yes |  | **-** | H2N2F1S1 *(su)* | No | Yes |
| D | H1N1F1 | Yes | No |  | **AA** | H3N3F1 | Yes | Yes |
| E | H2N1 | Yes | No |  | **AB** | H2N4F1 | Yes | No |
| F | H1N2 | Yes | Yes |  | **AC** | H3N4 | Yes | No |
| G | H1N1S1 | Yes | Yes |  | **AD** | H2N2S2 | Yes | Yes |
| H | H2N1F1 | Yes | No |  | **AE** | H2N3F1S1 | Yes | No |
| I | N2S1 | Yes | Yes |  | **AF** | H3N3F2 | Yes | No |
| J | H1N2F1 | Yes | No |  | **AG** | H3N3S1 | Yes | No |
| K | H2N2 | Yes | Yes |  | **AH** | H3N4F1 | Yes | No |
| L | H1N3 | Yes | No |  | **-** | H2N2F1S2 | No | Yes |
| M | H1N2S1 | Yes | Yes |  | **AI** | H4N4 | Yes | No |
| N | H2N2F1 | Yes | Yes |  | **AJ** | H3N2F2S1 | Yes | No |
| O | H3N2 | Yes | No |  | **AK** | H3N3F1S1 | Yes | No |
| - | H2N2F1 *(su)* | No | Yes |  | **AL** | H2N4F1S1 | Yes | No |
| P | H2N3 | Yes | No |  | **AM** | H3N4F2 | Yes | No |
| Q | H1N1S2 | Yes | Yes |  | **AN** | H4N4F1 | Yes | No |
| R | H1N4 | Yes | No |  | **AO** | H3N3F2S1 | Yes | No |
| S | H2N1F1S1 | Yes | No |  | **AP** | H3N3S2 | Yes | No |
| T | H2N2S1 | Yes | Yes |  | **AQ** | H3N4F1S1 | Yes | No |
| U | H3N2F1 | Yes | No |  | **AR** | H4N4F2 | Yes | No |
| V | H2N3F1 | Yes | No |  | **AS** | H4N4S1 | Yes | No |
| W | H3N3 | Yes | Yes |  | **AT** | H3N3F1S2 | Yes | No |
| X | H2N4 | Yes | No |  |  |  |  |  |

**Table 6.** Comparison of the *O*-glycan species identified from the glycosylation analysis of colorectal cancer cell line PaTu T with previously reported *O*-glycan species from the literature. *O*-glycan compositions detected in this study and/or previously reported in the literature are highlighted in green. *O*-glycan compositions not detected in this study and/or not reported in the literature are highlighted in red [2]. Glycan compositions are given in the terms of hexose (H), *N*-acetylhexosamine (N), deoxyhexose (F), *N*-acetylneuraminic acid (S), *N*-glycolylneuraminic acid (Sg), sulphated structure (*su*).

| PaTu T *O*-glycans | | | |  |
| --- | --- | --- | --- | --- |
| Peak ID | Composition | Detected (in this work) | Literature[1] (previously reported) |  |
| A | N1 | Yes | No |  |
| B | H2N1 | Yes | No |  |
| C | H1N1S1 | Yes | Yes |  |
| D | H2N2 | Yes | No |  |
| E | H1N2S1 | Yes | Yes |  |
| F | H2N2F1 | Yes | No |  |
| G | H3N2 | Yes | No |  |
| H | H1N1S2 | Yes | Yes |  |
| I | H2N2S1 | Yes | Yes |  |
| J | H3N2F1 | Yes | No |  |
| - | N2H2F1S1 | No | Yes |  |
| K | H3N3F1 | Yes | No |  |
| L | H2N2S2 | Yes | Yes |  |

**Table 7.** Comparison of the *O*-glycan species identified from the glycosylation analysis of colorectal cancer cell line PaTu S with previously reported *O*-glycan species from the literature. *O*-glycan compositions detected in this study and/or previously reported in the literature are highlighted in green. *O*-glycan compositions not detected in this study and/or not reported in the literature are highlighted in red [2]. Glycan compositions are given in the terms of hexose (H), *N*-acetylhexosamine (N), deoxyhexose (F), *N*-acetylneuraminic acid (S), *N*-glycolylneuraminic acid (Sg), sulphated structure (*su*).

| PaTu S *O*-glycans | | | |  | PaTu S *O*-glycans | | | |
| --- | --- | --- | --- | --- | --- | --- | --- | --- |
| Peak ID | Composition | Detected (in this work) | Literature[2] (previously reported) |  | Peak ID | Composition | Detected (in this work) | Literature[2] (previously reported |
| - | H1N1 | No | Yes |  | **X** | H3N4 | Yes | No |
| A | N2 | Yes | No |  | **Y** | H2N2S2 | Yes | No |
| B | N1S1 | Yes | Yes |  | **Z** | H2N5 | Yes | No |
| C | H2N1 | Yes | No |  | **AA** | H2N3F1S1 | Yes | Yes |
| D | H1N2 | Yes | Yes |  | **AB** | H3N3S1 | Yes | No |
| E | H1N1S1 | Yes | Yes |  | **AC** | H3N4F1 | Yes | No |
| F | H2N2 | Yes | Yes |  | **AD** | H4N4 | Yes | Yes |
| G | H1N3 | Yes | No |  | **AE** | H3N5 | Yes | No |
| H | H1N2S1 | Yes | Yes |  | **AF** | H4N4F1 | Yes | No |
| I | H2N2F1 | Yes | Yes |  | **AG** | H3N5F1 | Yes | No |
| J | H3N2 | Yes | No |  | **AH** | H4N5 | Yes | No |
| K | H1N3F1 | Yes | No |  | **AI** | H4N4F2 | Yes | No |
| L | H2N3 | Yes | No |  | **AJ** | H4N4S1 | Yes | No |
| M | H1N1S2 | Yes | Yes |  | **-** | H2N3F2S1 | No | Yes |
| N | H1N4 | Yes | Yes |  | **AK** | H4N5F1 | Yes | No |
| O | H2N2S1 | Yes | Yes |  | **AL** | H5N5 | Yes | No |
| P | H2N3F1 | Yes | Yes |  | **AM** | H4N6 | Yes | No |
| Q | H3N3 | Yes | Yes |  |  |  |  |  |
| R | H2N4 | Yes | Yes |  |  |  |  |  |
| S | H2N2F1S1 | Yes | Yes |  |  |  |  |  |
| - | H1N3F1S1 | No | Yes |  |  |  |  |  |
| T | H2N3F2 | Yes | No |  |  |  |  |  |
| U | H3N3F1 | Yes | No |  |  |  |  |  |
| V | H4N3 | Yes | No |  |  |  |  |  |
| W | H2N4F1 | Yes | No |  |  |  |  |  |

**Table 8.** SW480 human colorectal cancer cell line *O*-glycan identification and quantitation. Glycan compositions, calculated *m/z* values, average registered *m/z* values from three sample replicates (Avg. *m/z*), relative areas, mass precision (PPM), index of peak quality (IPQ), signal-to-noise (S/N) ratios, standard deviations (SDs) and coefficients of variation (CVs) for quantified *O*-glycan structures detected in SW480 human colorectal cancer cell line and calculated after triplicate analysis. Glycan compositions are given in the terms of hexose (H), *N*-acetylhexosamine (N), deoxyhexose (F), *N*-acetylneuraminic acid (S).

| Peak ID | Composition | | | | *m/z*  (calculated) | Avg *m/z*  (registered) | Criteria | SW480 *O*-glycan data | | | Average | Variation | | |  |
| --- | --- | --- | --- | --- | --- | --- | --- | --- | --- | --- | --- | --- | --- | --- | --- |
|  | Hex (H) | HexNAc (N) | Fuc (F) | Neu5Ac (S) |  |  |  | Replicate A | Replicate B | Replicate C |  | SD | | **CV** |  |
| A | 0 | 1 | 0 | 1 | 691.3624 | 691.3612 | Relative % area | 0.45 | 0.52 | 0.49 | 0.48 | 0.03 | | **6.32** |  |
|  |  |  |  |  |  |  | Mass precision (PPM) | 1.55 | 1.59 | 1.98 | 1.70 |  |  |  |  |
|  |  |  |  |  |  |  | Index of peak quality (IPQ) | 0.18 | 0.33 | 0.15 | 0.22 |  |  |  |  |
|  |  |  |  |  |  |  | Signal-to-noise ratio (S/N) | 19.84 | 16.12 | 19.61 | 18.52 |  |  |  |  |
| B | 2 | 1 | 0 | 0 | 738.3883 | 738.3867 | Relative % area | 7.73 | 11.37 | 11.90 | 10.33 | 1.86 | | **17.97** |  |
|  |  |  |  |  |  |  | Mass precision (PPM) | 2.29 | 2.16 | 2.02 | 2.16 |  |  |  |  |
|  |  |  |  |  |  |  | Index of peak quality (IPQ) | 0.00 | 0.01 | 0.08 | 0.03 |  |  |  |  |
|  |  |  |  |  |  |  | Signal-to-noise ratio (S/N) | 156.49 | 77.74 | 143.20 | 125.81 |  |  |  |  |
| C | 1 | 1 | 0 | 1 | 895.4621 | 895.4612 | Relative % area | 6.78 | 8.40 | 9.14 | 8.11 | 0.98 | | **12.14** |  |
|  |  |  |  |  |  |  | Mass precision (PPM) | 1.13 | 0.81 | 1.24 | 1.06 |  |  |  |  |
|  |  |  |  |  |  |  | Index of peak quality (IPQ) | 0.08 | 0.10 | 0.12 | 0.10 |  |  |  |  |
|  |  |  |  |  |  |  | Signal-to-noise ratio (S/N) | 50.00 | 85.27 | 66.35 | 67.21 |  |  |  |  |
| D | 2 | 2 | 0 | 0 | 983.5146 | 983.5145 | Relative % area | 31.79 | 35.00 | 32.73 | 33.17 | 1.35 | | **4.06** |  |
|  |  |  |  |  |  |  | Mass precision (PPM) | 0.07 | 0.03 | 0.06 | 0.06 |  |  |  |  |
|  |  |  |  |  |  |  | Index of peak quality (IPQ) | 0.06 | 0.03 | 0.04 | 0.05 |  |  |  |  |
|  |  |  |  |  |  |  | Signal-to-noise ratio (S/N) | 791.13 | 361.03 | 427.81 | 526.66 |  |  |  |  |
| E | 2 | 2 | 1 | 0 | 1157.6038 | 1157.6020 | Relative % area | 11.84 | 17.49 | 16.36 | 15.23 | 2.44 | | **16.03** |  |
|  |  |  |  |  |  |  | Mass precision (PPM) | 1.54 | 1.79 | 1.37 | 1.57 |  |  |  |  |
|  |  |  |  |  |  |  | Index of peak quality (IPQ) | 0.06 | 0.07 | 0.07 | 0.07 |  |  |  |  |
|  |  |  |  |  |  |  | Signal-to-noise ratio (S/N) | 225.74 | 158.37 | 158.13 | 180.75 |  |  |  |  |
| F | 3 | 2 | 0 | 0 | 1187.6144 | 1187.6121 | Relative % area | 8.06 | 7.68 | 6.47 | 7.40 | 0.68 | | **9.17** |  |
|  |  |  |  |  |  |  | Mass precision (PPM) | 2.12 | 2.03 | 1.62 | 1.92 |  |  |  |  |
|  |  |  |  |  |  |  | Index of peak quality (IPQ) | 0.06 | 0.04 | 0.12 | 0.07 |  |  |  |  |
|  |  |  |  |  |  |  | Signal-to-noise ratio (S/N) | 137.97 | 60.76 | 71.88 | 90.20 |  |  |  |  |
| G | 2 | 3 | 0 | 0 | 1228.6409 | 1228.6377 | Relative % area | 6.09 | 4.84 | 5.16 | 5.36 | 0.53 | | **9.87** |  |
|  |  |  |  |  |  |  | Mass precision (PPM) | 2.95 | 2.55 | 2.25 | 2.58 |  |  |  |  |
|  |  |  |  |  |  |  | Index of peak quality (IPQ) | 0.14 | 0.13 | 0.34 | 0.20 |  |  |  |  |
|  |  |  |  |  |  |  | Signal-to-noise ratio (S/N) | 44.50 | 40.92 | 54.04 | 46.49 |  |  |  |  |
| H | 1 | 1 | 0 | 2 | 1256.6358 | 1256.6322 | Relative % area | 4.80 | 4.84 | 6.88 | 5.51 | 0.97 | | **17.63** |  |
|  |  |  |  |  |  |  | Mass precision (PPM) | 3.39 | 2.51 | 2.74 | 2.88 |  |  |  |  |
|  |  |  |  |  |  |  | Index of peak quality (IPQ) | 0.17 | 0.26 | 0.16 | 0.20 |  |  |  |  |
|  |  |  |  |  |  |  | Signal-to-noise ratio (S/N) | 46.74 | 42.30 | 54.44 | 47.83 |  |  |  |  |
| I | 2 | 2 | 0 | 1 | 1344.6882 | 1344.6831 | Relative % area | 22.46 | 9.86 | 10.86 | 14.40 | 5.72 | **39.72** | | |
|  |  |  |  |  |  |  | Mass precision (PPM) | 3.90 | 4.12 | 3.49 | 3.84 |  |  |  |  |
|  |  |  |  |  |  |  | Index of peak quality (IPQ) | 0.13 | 0.13 | 0.25 | 0.17 |  |  |  |  |
|  |  |  |  |  |  |  | Signal-to-noise ratio (S/N) | 224.91 | 63.45 | 76.83 | 121.73 |  |  |  |  |

**Table 9.** SW620 human colorectal cancer cell line *O*-glycan identification and quantitation. Glycan compositions, calculated *m/z* values, average registered *m/z* values from three sample replicates (Avg. *m/z*), relative areas, mass precision (PPM), index of peak quality (IPQ), signal-to-noise (S/N) ratios, standard deviations (SDs) and coefficients of variation (CVs) for quantified *O*-glycan structures detected in SW620 human colorectal cancer cell line and calculated after triplicate analysis. Glycan compositions are given in the terms of hexose (H), *N*-acetylhexosamine (N), deoxyhexose (F), *N*-acetylneuraminic acid (S).

| Peak ID | Composition | | | | *m/z*  (calculated) | Avg *m/z*  (registered) | Criteria | SW620 *O*-glycan data | | | Average | Variation | | | |
| --- | --- | --- | --- | --- | --- | --- | --- | --- | --- | --- | --- | --- | --- | --- | --- |
|  |  |  |  |  |  |  |  | Replicate A | Replicate B | Replicate C |  | SD | **CV** | | |
|  | Hex (H) | HexNAc (N) | Fuc (F) | Neu5Ac (S) |  |  |  |  |  |  |  |  |  |  |  |
| A | 0 | 2 | 0 | 0 | 575.3150 | 575.3135 | Relative % area | 8.29 | 7.19 | 12.38 | 9.29 | 2.23 | **24.06** | | |
|  |  |  |  |  |  |  | Mass precision (PPM) | 2.78 | 2.13 | 2.82 | 2.58 |  |  |  |  |
|  |  |  |  |  |  |  | Index of peak quality (IPQ) | 0.18 | 0.21 | 0.11 | 0.17 |  |  |  |  |
|  |  |  |  |  |  |  | Signal-to-noise ratio (S/N) | 196.29 | 117.69 | 260.00 | 191.33 |  |  |  |  |
| B | 1 | 2 | 0 | 0 | 779.4148 | 779.4141 | Relative % area | 5.95 | 5.19 | 5.39 | 5.51 | 0.32 | **5.81** | | |
|  |  |  |  |  |  |  | Mass precision (PPM) | 1.04 | 1.12 | 0.61 | 0.93 |  |  |  |  |
|  |  |  |  |  |  |  | Index of peak quality (IPQ) | 0.12 | 0.17 | 0.06 | 0.12 |  |  |  |  |
|  |  |  |  |  |  |  | Signal-to-noise ratio (S/N) | 275.22 | 125.72 | 255.05 | 218.66 |  |  |  |  |
| C | 0 | 3 | 0 | 0 | 820.4414 | 820.4405 | Relative % area | 1.77 | 1.68 | 2.18 | 1.88 | 0.22 | **11.59** | | |
|  |  |  |  |  |  |  | Mass precision (PPM) | 0.90 | 1.14 | 1.14 | 1.06 |  |  |  |  |
|  |  |  |  |  |  |  | Index of peak quality (IPQ) | 0.10 | 0.38 | 0.22 | 0.23 |  |  |  |  |
|  |  |  |  |  |  |  | Signal-to-noise ratio (S/N) | 119.42 | 62.69 | 115.79 | 99.30 |  |  |  |  |
| D | 1 | 1 | 0 | 1 | 895.4621 | 895.4614 | Relative % area | 8.21 | 8.62 | 7.83 | 8.22 | 0.32 | **3.89** | | |
|  |  |  |  |  |  |  | Mass precision (PPM) | 0.85 | 0.80 | 0.80 | 0.82 |  |  |  |  |
|  |  |  |  |  |  |  | Index of peak quality (IPQ) | 0.18 | 0.14 | 0.19 | 0.17 |  |  |  |  |
|  |  |  |  |  |  |  | Signal-to-noise ratio (S/N) | 349.94 | 305.62 | 362.03 | 339.20 |  |  |  |  |
| E | 1 | 2 | 1 | 0 | 953.5040 | 953.5037 | Relative % area | 1.14 | 1.35 | 0.88 | 1.13 | 0.19 | **17.14** | | |
|  |  |  |  |  |  |  | Mass precision (PPM) | 0.07 | 0.44 | 0.44 | 0.32 |  |  |  |  |
|  |  |  |  |  |  |  | Index of peak quality (IPQ) | 0.26 | 0.22 | 0.21 | 0.23 |  |  |  |  |
|  |  |  |  |  |  |  | Signal-to-noise ratio (S/N) | 34.71 | 23.34 | 33.37 | 30.47 |  |  |  |  |
| F | 2 | 2 | 0 | 0 | 983.5146 | 983.5145 | Relative % area | 8.17 | 7.45 | 7.57 | 7.73 | 0.32 | **4.08** | | |
|  |  |  |  |  |  |  | Mass precision (PPM) | 0.10 | 0.01 | 0.01 | 0.04 |  |  |  |  |
|  |  |  |  |  |  |  | Index of peak quality (IPQ) | 0.09 | 0.04 | 0.06 | 0.06 |  |  |  |  |
|  |  |  |  |  |  |  | Signal-to-noise ratio (S/N) | 458.12 | 221.01 | 314.35 | 331.16 |  |  |  |  |
| G | 1 | 2 | 0 | 1 | 1140.5885 | 1140.5870 | Relative % area | 1.71 | 2.06 | 2.20 | 1.99 | 0.21 | **10.39** | | |
|  |  |  |  |  |  |  | Mass precision (PPM) | 1.55 | 1.32 | 0.97 | 1.28 |  |  |  |  |
|  |  |  |  |  |  |  | Index of peak quality (IPQ) | 0.05 | 0.13 | 0.16 | 0.11 |  |  |  |  |
|  |  |  |  |  |  |  | Signal-to-noise ratio (S/N) | 67.78 | 46.09 | 81.70 | 65.19 |  |  |  |  |
| H | 2 | 2 | 1 | 0 | 1157.6038 | 1157.6027 | Relative % area | 2.53 | 2.30 | 2.33 | 2.38 | 0.10 | **4.29** | | |
|  |  |  |  |  |  |  | Mass precision (PPM) | 0.91 | 1.01 | 1.01 | 0.97 |  |  |  |  |
|  |  |  |  |  |  |  | Index of peak quality (IPQ) | 0.09 | 0.25 | 0.08 | 0.14 |  |  |  |  |
|  |  |  |  |  |  |  | Signal-to-noise ratio (S/N) | 107.59 | 44.57 | 85.55 | 79.24 |  |  |  |  |
| I | 3 | 2 | 0 | 0 | 1187.6144 | 1187.6129 | Relative % area | 3.12 | 3.29 | 2.63 | 3.01 | 0.28 | **9.36** | | |
|  |  |  |  |  |  |  | Mass precision (PPM) | 1.45 | 1.16 | 1.16 | 1.26 |  |  |  |  |
|  |  |  |  |  |  |  | Index of peak quality (IPQ) | 0.14 | 0.08 | 0.18 | 0.13 |  |  |  |  |
|  |  |  |  |  |  |  | Signal-to-noise ratio (S/N) | 96.93 | 58.80 | 86.67 | 80.80 |  |  |  |  |
| J | 2 | 3 | 0 | 0 | 1228.6409 | 1228.6387 | Relative % area | 3.31 | 3.60 | 2.83 | 3.25 | 0.31 | **9.68** | | |
|  |  |  |  |  |  |  | Mass precision (PPM) | 1.91 | 2.00 | 1.35 | 1.75 |  |  |  |  |
|  |  |  |  |  |  |  | Index of peak quality (IPQ) | 0.11 | 0.05 | 0.13 | 0.10 |  |  |  |  |
|  |  |  |  |  |  |  | Signal-to-noise ratio (S/N) | 54.53 | 53.43 | 71.68 | 59.88 |  |  |  |  |
| K | 1 | 1 | 0 | 2 | 1256.6358 | 1256.6332 | Relative % area | 13.71 | 13.91 | 13.46 | 13.69 | 0.19 | **1.36** | | |
|  |  |  |  |  |  |  | Mass precision (PPM) | 2.47 | 2.10 | 1.78 | 2.12 |  |  |  |  |
|  |  |  |  |  |  |  | Index of peak quality (IPQ) | 0.05 | 0.10 | 0.06 | 0.07 |  |  |  |  |
|  |  |  |  |  |  |  | Signal-to-noise ratio (S/N) | 300.53 | 256.40 | 397.42 | 318.12 |  |  |  |  |
| L | 2 | 2 | 0 | 1 | 1344.6882 | 1344.6847 | Relative % area | 12.74 | 13.53 | 13.28 | 13.18 | 0.33 | **2.48** | | |
|  |  |  |  |  |  |  | Mass precision (PPM) | 3.00 | 2.56 | 2.26 | 2.61 |  |  |  |  |
|  |  |  |  |  |  |  | Index of peak quality (IPQ) | 0.06 | 0.18 | 0.08 | 0.11 |  |  |  |  |
|  |  |  |  |  |  |  | Signal-to-noise ratio (S/N) | 237.11 | 213.19 | 314.58 | 254.96 |  |  |  |  |
| M | 3 | 3 | 0 | 0 | 1432.7407 | 1432.7360 | Relative % area | 7.45 | 7.48 | 7.28 | 7.40 | 0.09 | **1.22** | | |
|  |  |  |  |  |  |  | Mass precision (PPM) | 3.69 | 3.06 | 3.06 | 3.27 |  |  |  |  |
|  |  |  |  |  |  |  | Index of peak quality (IPQ) | 0.07 | 0.12 | 0.13 | 0.11 |  |  |  |  |
|  |  |  |  |  |  |  | Signal-to-noise ratio (S/N) | 96.67 | 83.01 | 130.91 | 103.53 |  |  |  |  |
| N | 1 | 2 | 0 | 2 | 1501.7621 | 1501.7573 | Relative % area | 3.33 | 2.93 | 3.11 | 3.12 | 0.16 | **5.25** | | |
|  |  |  |  |  |  |  | Mass precision (PPM) | 3.63 | 3.44 | 2.64 | 3.24 |  |  |  |  |
|  |  |  |  |  |  |  | Index of peak quality (IPQ) | 0.10 | 0.21 | 0.12 | 0.14 |  |  |  |  |
|  |  |  |  |  |  |  | Signal-to-noise ratio (S/N) | 62.24 | 29.96 | 59.52 | 50.57 |  |  |  |  |
| O | 2 | 3 | 0 | 1 | 1589.8146 | 1589.8093 | Relative % area | 3.13 | 4.89 | 4.10 | 4.04 | 0.72 | **17.78** | | |
|  |  |  |  |  |  |  | Mass precision (PPM) | 3.63 | 3.49 | 2.74 | 3.28 |  |  |  |  |
|  |  |  |  |  |  |  | Index of peak quality (IPQ) | 0.24 | 0.17 | 0.07 | 0.16 |  |  |  |  |
|  |  |  |  |  |  |  | Signal-to-noise ratio (S/N) | 56.06 | 31.08 | 55.57 | 47.57 |  |  |  |  |
| P | 3 | 3 | 1 | 0 | 1606.8299 | 1606.8229 | Relative % area | 4.27 | 2.57 | 2.71 | 3.18 | 0.77 | **24.13** | | |
|  |  |  |  |  |  |  | Mass precision (PPM) | 5.27 | 4.40 | 3.40 | 4.36 |  |  |  |  |
|  |  |  |  |  |  |  | Index of peak quality (IPQ) | 0.03 | 0.16 | 0.22 | 0.14 |  |  |  |  |
|  |  |  |  |  |  |  | Signal-to-noise ratio (S/N) | 36.89 | 13.81 | 30.33 | 27.01 |  |  |  |  |
| Q | 4 | 3 | 0 | 0 | 1636.8404 | 1636.8333 | Relative % area | 1.74 | 2.92 | 2.13 | 2.26 | 0.49 | **21.82** | | |
|  |  |  |  |  |  |  | Mass precision (PPM) | 5.52 | 3.89 | 3.64 | 4.35 |  |  |  |  |
|  |  |  |  |  |  |  | Index of peak quality (IPQ) | 0.19 | 0.41 | 0.12 | 0.24 |  |  |  |  |
|  |  |  |  |  |  |  | Signal-to-noise ratio (S/N) | 27.17 | 15.92 | 25.86 | 22.98 |  |  |  |  |
| R | 3 | 4 | 0 | 0 | 1677.8670 | 1677.8591 | Relative % area | 3.30 | 2.76 | 2.06 | 2.71 | 0.51 | **18.80** | | |
|  |  |  |  |  |  |  | Mass precision (PPM) | 5.46 | 3.84 | 4.79 | 4.70 |  |  |  |  |
|  |  |  |  |  |  |  | Index of peak quality (IPQ) | 0.14 | 0.36 | 0.05 | 0.18 |  |  |  |  |
|  |  |  |  |  |  |  | Signal-to-noise ratio (S/N) | 42.23 | 22.41 | 26.83 | 30.49 |  |  |  |  |
| S | 2 | 2 | 0 | 2 | 1705.8619 | 1705.8545 | Relative % area | 1.27 | 1.08 | 0.93 | 1.10 | 0.14 | **12.75** | | |
|  |  |  |  |  |  |  | Mass precision (PPM) | 4.78 | 4.68 | 3.50 | 4.32 |  |  |  |  |
|  |  |  |  |  |  |  | Index of peak quality (IPQ) | 0.15 | 0.25 | 0.25 | 0.22 |  |  |  |  |
|  |  |  |  |  |  |  | Signal-to-noise ratio (S/N) | 18.33 | 8.80 | 14.18 | 13.77 |  |  |  |  |
|  |  |  |  |  |  |  |  |  |  |  |  |  | |  |  |
|  |  |  |  |  |  |  |  |  |  |  |  |  | |  |  |
| T | 3 | 3 | 0 | 1 | 1793.9143 | 1793.9062 | Relative % area | 4.85 | 5.18 | 4.73 | 4.92 | 0.19 | | **3.92** |  |
|  |  |  |  |  |  |  | Mass precision (PPM) | 5.15 | 4.71 | 3.82 | 4.56 |  |  |  |  |
|  |  |  |  |  |  |  | Index of peak quality (IPQ) | 0.11 | 0.08 | 0.26 | 0.15 |  |  |  |  |
|  |  |  |  |  |  |  | Signal-to-noise ratio (S/N) | 59.88 | 31.03 | 54.95 | 48.62 |  |  |  |  |

**Table 10.** LS174T human colorectal cancer cell line *O*-glycan identification and quantitation. Glycan compositions, calculated *m/z* values, average registered *m/z* values from three sample replicates (Avg. *m/z*), relative areas, mass precision (PPM), index of peak quality (IPQ), signal-to-noise (S/N) ratios, standard deviations (SDs) and coefficients of variation (CVs) for quantified *O*-glycan structures detected in LS174T human colorectal cancer cell line and calculated after triplicate analysis. Glycan compositions are given in the terms of hexose (H), *N*-acetylhexosamine (N), deoxyhexose (F), *N*-acetylneuraminic acid (S).

| Peak ID | Composition | | | | *m/z*  (calculated) | Avg *m/z*  (registered) | | Criteria | LS174T *O*-glycan data | | | Average | Variation | |
| --- | --- | --- | --- | --- | --- | --- | --- | --- | --- | --- | --- | --- | --- | --- |
|  | Hex (H) | HexNAc (N) | Fuc (F) | Neu5Ac (S) |  |  |  |  | Replicate A | Replicate B | Replicate C |  | SD | **CV** |
| A | 1 | 1 | 0 | 0 | 534.2885 | 534.2883 | | Relative % area | 1.2928 | 1.5227 | 1.8053 | 1.5403 | 0.21 | **13.61** |
|  |  |  |  |  |  |  |  | Mass precision (PPM) | 0.38 | 0.53 | 0.15 | 0.36 |  |  |
|  |  |  |  |  |  |  |  | Index of peak quality (IPQ) | 0.18 | 0.14 | 0.17 | 0.16 |  |  |
|  |  |  |  |  |  |  |  | Signal-to-noise ratio (S/N) | 955.20 | 1028.24 | 547.55 | 843.66 |  |  |
| B | 0 | 2 | 0 | 0 | 575.3150 | 575.3148 | | Relative % area | 0.1211 | 0.1628 | 0.2229 | 0.1689 | 0.04 | **24.73** |
|  |  |  |  |  |  |  |  | Mass precision (PPM) | 0.4294 | 0.4063 | 0.1583 | 0.3313 |  |  |
|  |  |  |  |  |  |  |  | Index of peak quality (IPQ) | 0.18 | 0.17 | 0.19 | 0.18 |  |  |
|  |  |  |  |  |  |  |  | Signal-to-noise ratio (S/N) | 78.07 | 101.27 | 63.44 | 80.92 |  |  |
| C | 0 | 1 | 0 | 1 | 691.3624 | 691.3619 | | Relative % area | 0.8264 | 1.0166 | 1.0610 | 0.9680 | 0.10 | **10.51** |
|  |  |  |  |  |  |  |  | Mass precision (PPM) | 0.83 | 0.76 | 0.57 | 0.72 |  |  |
|  |  |  |  |  |  |  |  | Index of peak quality (IPQ) | 0.14 | 0.12 | 0.14 | 0.13 |  |  |
|  |  |  |  |  |  |  |  | Signal-to-noise ratio (S/N) | 457.94 | 545.88 | 362.10 | 455.30 |  |  |
| D | 1 | 1 | 1 | 0 | 708.3777 | 708.3771 | | Relative % area | 0.1803 | 0.1894 | 0.2570 | 0.2089 | 0.03 | **16.38** |
|  |  |  |  |  |  |  |  | Mass precision (PPM) | 1.00 | 0.96 | 0.75 | 0.90 |  |  |
|  |  |  |  |  |  |  |  | Index of peak quality (IPQ) | 0.22 | 0.09 | 0.27 | 0.20 |  |  |
|  |  |  |  |  |  |  |  | Signal-to-noise ratio (S/N) | 133.71 | 216.53 | 106.50 | 152.25 |  |  |
| E | 2 | 1 | 0 | 0 | 738.3883 | 738.3879 | | Relative % area | 0.5530 | 0.4782 | 0.5705 | 0.5339 | 0.04 | **7.50** |
|  |  |  |  |  |  |  |  | Mass precision (PPM) | 0.79 | 0.23 | 0.54 | 0.52 |  |  |
|  |  |  |  |  |  |  |  | Index of peak quality (IPQ) | 0.07 | 0.07 | 0.14 | 0.09 |  |  |
|  |  |  |  |  |  |  |  | Signal-to-noise ratio (S/N) | 379.31 | 487.46 | 319.28 | 395.35 |  |  |
| F | 1 | 2 | 0 | 0 | 779.4148 | 779.4142 | | Relative % area | 1.0585 | 1.1063 | 1.3683 | 1.1777 | 0.14 | **11.56** |
|  |  |  |  |  |  |  |  | Mass precision (PPM) | 0.63 | 0.66 | 1.03 | 0.77 |  |  |
|  |  |  |  |  |  |  |  | Index of peak quality (IPQ) | 0.06 | 0.04 | 0.03 | 0.04 |  |  |
|  |  |  |  |  |  |  |  | Signal-to-noise ratio (S/N) | 888.91 | 1287.97 | 751.15 | 976.01 |  |  |
| G | 1 | 1 | 0 | 1 | 895.4621 | 895.4621 | | Relative % area | 4.4361 | 4.8339 | 4.8510 | 4.7070 | 0.19 | **4.07** |
|  |  |  |  |  |  |  |  | Mass precision (PPM) | 0.19 | 0.02 | 0.05 | 0.09 |  |  |
|  |  |  |  |  |  |  |  | Index of peak quality (IPQ) | 0.06 | 0.05 | 0.04 | 0.05 |  |  |
|  |  |  |  |  |  |  |  | Signal-to-noise ratio (S/N) | 2695.81 | 3307.45 | 1917.09 | 2640.12 |  |  |
| H | 2 | 1 | 1 | 0 | 912.4775 | 912.4768 | | Relative % area | 0.1599 | 0.1847 | 0.2208 | 0.1885 | 0.02 | **13.26** |
|  |  |  |  |  |  |  |  | Mass precision (PPM) | 0.58 | 0.78 | 0.83 | 0.73 |  |  |
|  |  |  |  |  |  |  |  | Index of peak quality (IPQ) | 0.21 | 0.11 | 0.22 | 0.18 |  |  |
|  |  |  |  |  |  |  |  | Signal-to-noise ratio (S/N) | 118.24 | 159.73 | 96.50 | 124.82 |  |  |
| I | 0 | 2 | 0 | 1 | 936.4887 | 936.4885 | | Relative % area | 0.3122 | 0.3145 | 0.3100 | 0.3122 | 0.00 | **0.58** |
|  |  |  |  |  |  |  |  | Mass precision (PPM) | 0.37 | 0.17 | 0.19 | 0.24 |  |  |
|  |  |  |  |  |  |  |  | Index of peak quality (IPQ) | 0.10 | 0.10 | 0.18 | 0.13 |  |  |
|  |  |  |  |  |  |  |  | Signal-to-noise ratio (S/N) | 149.94 | 168.64 | 68.41 | 129.00 |  |  |
| J | 1 | 2 | 1 | 0 | 953.5040 | 953.5038 | | Relative % area | 0.2182 | 0.2429 | 0.3110 | 0.2573 | 0.04 | **15.24** |
|  |  |  |  |  |  |  |  | Mass precision (PPM) | 0.14 | 0.13 | 0.38 | 0.22 |  |  |
|  |  |  |  |  |  |  |  | Index of peak quality (IPQ) | 0.15 | 0.15 | 0.22 | 0.17 |  |  |
|  |  |  |  |  |  |  |  | Signal-to-noise ratio (S/N) | 127.65 | 166.29 | 82.32 | 125.42 |  |  |
| K | 2 | 2 | 0 | 0 | 983.5146 | 983.5145 | | Relative % area | 5.6827 | 5.7391 | 6.2612 | 5.8943 | 0.26 | **4.42** |
|  |  |  |  |  |  |  |  | Mass precision (PPM) | 0.23 | 0.07 | 0.02 | 0.11 |  |  |
|  |  |  |  |  |  |  |  | Index of peak quality (IPQ) | 0.05 | 0.06 | 0.05 | 0.05 |  |  |
|  |  |  |  |  |  |  |  | Signal-to-noise ratio (S/N) | 4120.02 | 4825.83 | 2410.49 | 3785.45 |  |  |
| L | 1 | 3 | 0 | 0 | 1024.5411 | 1024.5408 | | Relative % area | 0.3572 | 0.3694 | 0.3277 | 0.3514 | 0.02 | **4.97** |
|  |  |  |  |  |  |  |  | Mass precision (PPM) | 0.07 | 0.42 | 0.33 | 0.27 |  |  |
|  |  |  |  |  |  |  |  | Index of peak quality (IPQ) | 0.10 | 0.09 | 0.21 | 0.13 |  |  |
|  |  |  |  |  |  |  |  | Signal-to-noise ratio (S/N) | 248.20 | 219.10 | 118.60 | 195.30 |  |  |
| M | 1 | 2 | 0 | 1 | 1140.5885 | 1140.5879 | | Relative % area | 1.0154 | 1.0425 | 1.1101 | 1.0560 | 0.04 | **3.77** |
|  |  |  |  |  |  |  |  | Mass precision (PPM) | 0.67 | 0.40 | 0.39 | 0.49 |  |  |
|  |  |  |  |  |  |  |  | Index of peak quality (IPQ) | 0.14 | 0.13 | 0.12 | 0.13 |  |  |
|  |  |  |  |  |  |  |  | Signal-to-noise ratio (S/N) | 348.34 | 324.93 | 179.75 | 284.34 |  |  |
| N | 2 | 2 | 1 | 0 | 1157.6038 | 1157.6025 | | Relative % area | 2.8096 | 2.7267 | 2.7855 | 2.7739 | 0.03 | **1.25** |
|  |  |  |  |  |  |  |  | Mass precision (PPM) | 1.13 | 1.25 | 0.84 | 1.07 |  |  |
|  |  |  |  |  |  |  |  | Index of peak quality (IPQ) | 0.05 | 0.04 | 0.04 | 0.04 |  |  |
|  |  |  |  |  |  |  |  | Signal-to-noise ratio (S/N) | 1206.04 | 1360.21 | 589.15 | 1051.80 |  |  |
| O | 3 | 2 | 0 | 0 | 1187.6144 | 1187.6134 | | Relative % area | 1.2666 | 1.3490 | 1.5310 | 1.3822 | 0.11 | **7.99** |
|  |  |  |  |  |  |  |  | Mass precision (PPM) | 1.22 | 0.62 | 0.58 | 0.81 |  |  |
|  |  |  |  |  |  |  |  | Index of peak quality (IPQ) | 0.01 | 0.03 | 0.03 | 0.02 |  |  |
|  |  |  |  |  |  |  |  | Signal-to-noise ratio (S/N) | 594.91 | 636.87 | 362.13 | 531.31 |  |  |
| P | 2 | 3 | 0 | 0 | 1228.6409 | 1228.6398 | | Relative % area | 1.7313 | 1.6543 | 1.7864 | 1.7240 | 0.05 | **3.14** |
|  |  |  |  |  |  |  |  | Mass precision (PPM) | 1.10 | 0.85 | 0.78 | 0.91 |  |  |
|  |  |  |  |  |  |  |  | Index of peak quality (IPQ) | 0.02 | 0.02 | 0.06 | 0.03 |  |  |
|  |  |  |  |  |  |  |  | Signal-to-noise ratio (S/N) | 835.47 | 843.15 | 478.98 | 719.20 |  |  |
| Q | 1 | 1 | 0 | 2 | 1256.6358 | 1256.6348 | | Relative % area | 1.2341 | 1.3261 | 1.1680 | 1.2427 | 0.06 | **5.22** |
|  |  |  |  |  |  |  |  | Mass precision (PPM) | 1.08 | 0.68 | 0.74 | 0.83 |  |  |
|  |  |  |  |  |  |  |  | Index of peak quality (IPQ) | 0.11 | 0.05 | 0.11 | 0.09 |  |  |
|  |  |  |  |  |  |  |  | Signal-to-noise ratio (S/N) | 470.87 | 616.82 | 258.47 | 448.72 |  |  |
| R | 1 | 4 | 0 | 0 | 1269.6675 | 1269.6659 | | Relative % area | 0.3987 | 0.4128 | 0.4104 | 0.4073 | 0.01 | **1.51** |
|  |  |  |  |  |  |  |  | Mass precision (PPM) | 1.71 | 1.32 | 0.74 | 1.26 |  |  |
|  |  |  |  |  |  |  |  | Index of peak quality (IPQ) | 0.05 | 0.08 | 0.08 | 0.07 |  |  |
|  |  |  |  |  |  |  |  | Signal-to-noise ratio (S/N) | 98.15 | 150.89 | 66.66 | 105.23 |  |  |
| S | 2 | 1 | 1 | 1 | 1273.6511 | | 1273.6490 | Relative % area | 0.4686 | 0.4915 | 0.5190 | 0.4930 | 0.02 | **4.18** |
|  |  |  |  |  |  |  |  | Mass precision (PPM) | 2.12 | 1.42 | 1.46 | 1.67 |  |  |
|  |  |  |  |  |  |  |  | Index of peak quality (IPQ) | 0.27 | 0.07 | 0.30 | 0.21 |  |  |
|  |  |  |  |  |  |  |  | Signal-to-noise ratio (S/N) | 103.76 | 211.46 | 80.08 | 131.77 |  |  |
|  |  |  |  |  |  | |  |  |  |  |  |  |  |  |
|  |  |  |  |  |  | |  |  |  |  |  |  |  |  |
| T | 2 | 2 | 0 | 1 | 1344.6882 | | 1344.6862 | Relative % area | 7.5941 | 8.1278 | 8.1667 | 7.9629 | 0.26 | **3.28** |
|  |  |  |  |  |  |  |  | Mass precision (PPM) | 1.87 | 1.28 | 1.49 | 1.55 |  |  |
|  |  |  |  |  |  |  |  | Index of peak quality (IPQ) | 0.04 | 0.04 | 0.03 | 0.04 |  |  |
|  |  |  |  |  |  |  |  | Signal-to-noise ratio (S/N) | 1359.15 | 1505.94 | 939.82 | 1268.31 |  |  |
| U | 3 | 2 | 1 | 0 | 1361.7036 | | 1361.7009 | Relative % area | 1.0797 | 1.1247 | 1.0033 | 1.0692 | 0.05 | **4.69** |
|  |  |  |  |  |  |  |  | Mass precision (PPM) | 2.23 | 1.86 | 1.84 | 1.98 |  |  |
|  |  |  |  |  |  |  |  | Index of peak quality (IPQ) | 0.06 | 0.07 | 0.06 | 0.06 |  |  |
|  |  |  |  |  |  |  |  | Signal-to-noise ratio (S/N) | 205.29 | 207.99 | 132.20 | 181.83 |  |  |
| V | 2 | 3 | 1 | 0 | 1402.7301 | | 1402.7278 | Relative % area | 0.9729 | 0.9883 | 0.9659 | 0.9757 | 0.01 | **0.96** |
|  |  |  |  |  |  |  |  | Mass precision (PPM) | 2.11 | 1.41 | 1.41 | 1.64 |  |  |
|  |  |  |  |  |  |  |  | Index of peak quality (IPQ) | 0.06 | 0.10 | 0.12 | 0.09 |  |  |
|  |  |  |  |  |  |  |  | Signal-to-noise ratio (S/N) | 284.54 | 336.20 | 127.72 | 249.49 |  |  |
| W | 3 | 3 | 0 | 0 | 1432.7407 | | 1432.7378 | Relative % area | 6.9514 | 6.6741 | 6.6714 | 6.7656 | 0.13 | **1.94** |
|  |  |  |  |  |  |  |  | Mass precision (PPM) | 2.37 | 1.73 | 1.95 | 2.02 |  |  |
|  |  |  |  |  |  |  |  | Index of peak quality (IPQ) | 0.03 | 0.03 | 0.02 | 0.03 |  |  |
|  |  |  |  |  |  |  |  | Signal-to-noise ratio (S/N) | 2165.40 | 2232.60 | 918.72 | 1772.24 |  |  |
| X | 2 | 4 | 0 | 0 | 1473.7672 | | 1473.7636 | Relative % area | 1.4351 | 1.3157 | 1.4606 | 1.4038 | 0.06 | **4.50** |
|  |  |  |  |  |  |  |  | Mass precision (PPM) | 2.82 | 2.46 | 2.10 | 2.46 |  |  |
|  |  |  |  |  |  |  |  | Index of peak quality (IPQ) | 0.05 | 0.06 | 0.09 | 0.07 |  |  |
|  |  |  |  |  |  |  |  | Signal-to-noise ratio (S/N) | 322.09 | 367.35 | 160.24 | 283.23 |  |  |
| Y | 2 | 2 | 1 | 1 | 1518.7775 | | 1518.7727 | Relative % area | 12.7200 | 12.8084 | 12.1601 | 12.5628 | 0.29 | **2.28** |
|  |  |  |  |  |  |  |  | Mass precision (PPM) | 3.63 | 2.87 | 2.89 | 3.13 |  |  |
|  |  |  |  |  |  |  |  | Index of peak quality (IPQ) | 0.01 | 0.02 | 0.02 | 0.02 |  |  |
|  |  |  |  |  |  |  |  | Signal-to-noise ratio (S/N) | 1320.33 | 1244.68 | 565.47 | 1043.49 |  |  |
| Z | 3 | 2 | 2 | 0 | 1535.7928 | | 1535.7855 | Relative % area | 0.5889 | 0.6189 | 0.6027 | 0.6035 | 0.01 | **2.04** |
|  |  |  |  |  |  |  |  | Mass precision (PPM) | 4.99 | 4.63 | 4.50 | 4.71 |  |  |
|  |  |  |  |  |  |  |  | Index of peak quality (IPQ) | 0.17 | 0.24 | 0.29 | 0.23 |  |  |
|  |  |  |  |  |  |  |  | Signal-to-noise ratio (S/N) | 63.79 | 70.19 | 34.14 | 56.04 |  |  |
| AA | 3 | 3 | 1 | 0 | 1606.8299 | | 1606.8244 | Relative % area | 4.4892 | 4.4919 | 4.0191 | 4.3334 | 0.22 | **5.13** |
|  |  |  |  |  |  |  |  | Mass precision (PPM) | 3.97 | 3.08 | 3.17 | 3.41 |  |  |
|  |  |  |  |  |  |  |  | Index of peak quality (IPQ) | 0.06 | 0.07 | 0.05 | 0.06 |  |  |
|  |  |  |  |  |  |  |  | Signal-to-noise ratio (S/N) | 707.85 | 944.44 | 376.18 | 676.16 |  |  |
| AB | 2 | 4 | 1 | 0 | 1647.8564 | | 1647.8493 | Relative % area | 0.4220 | 0.4076 | 0.4230 | 0.4175 | 0.01 | **1.69** |
|  |  |  |  |  |  |  |  | Mass precision (PPM) | 4.87 | 4.12 | 4.06 | 4.35 |  |  |
|  |  |  |  |  |  |  |  | Index of peak quality (IPQ) | 0.06 | 0.05 | 0.15 | 0.09 |  |  |
|  |  |  |  |  |  |  |  | Signal-to-noise ratio (S/N) | 36.17 | 49.96 | 41.76 | 42.63 |  |  |
| AC | 3 | 4 | 0 | 0 | 1677.8670 | | 1677.8601 | Relative % area | 3.5256 | 3.4842 | 3.5103 | 3.5067 | 0.02 | **0.49** |
|  |  |  |  |  |  |  |  | Mass precision (PPM) | 4.78 | 3.83 | 3.73 | 4.11 |  |  |
|  |  |  |  |  |  |  |  | Index of peak quality (IPQ) | 0.05 | 0.05 | 0.04 | 0.04 |  |  |
|  |  |  |  |  |  |  |  | Signal-to-noise ratio (S/N) | 649.22 | 819.73 | 346.91 | 605.29 |  |  |
| AD | 2 | 2 | 0 | 2 | 1705.8619 | | 1705.8540 | Relative % area | 3.0050 | 3.4360 | 3.4176 | 3.2862 | 0.20 | **6.05** |
|  |  |  |  |  |  |  |  | Mass precision (PPM) | 5.24 | 4.59 | 4.17 | 4.66 |  |  |
|  |  |  |  |  |  |  |  | Index of peak quality (IPQ) | 0.06 | 0.06 | 0.07 | 0.06 |  |  |
|  |  |  |  |  |  |  |  | Signal-to-noise ratio (S/N) | 339.85 | 508.10 | 306.44 | 384.80 |  |  |
| AE | 2 | 3 | 1 | 1 | 1763.9038 | | 1763.8942 | Relative % area | 1.2162 | 1.2296 | 1.0895 | 1.1784 | 0.06 | **5.35** |
|  |  |  |  |  |  |  |  | Mass precision (PPM) | 6.27 | 4.85 | 5.17 | 5.43 |  |  |
|  |  |  |  |  |  |  |  | Index of peak quality (IPQ) | 0.21 | 0.18 | 0.19 | 0.19 |  |  |
|  |  |  |  |  |  |  |  | Signal-to-noise ratio (S/N) | 90.96 | 123.72 | 63.86 | 92.85 |  |  |
| AF | 3 | 3 | 2 | 0 | 1780.9191 | | 1780.9096 | Relative % area | 1.5956 | 1.5720 | 1.3663 | 1.5113 | 0.10 | **6.82** |
|  |  |  |  |  |  |  |  | Mass precision (PPM) | 6.09 | 5.12 | 4.79 | 5.33 |  |  |
|  |  |  |  |  |  |  |  | Index of peak quality (IPQ) | 0.08 | 0.07 | 0.06 | 0.07 |  |  |
|  |  |  |  |  |  |  |  | Signal-to-noise ratio (S/N) | 118.52 | 148.90 | 80.24 | 115.89 |  |  |
| AG | 3 | 3 | 0 | 1 | 1793.9143 | | 1793.9052 | Relative % area | 2.7480 | 3.1000 | 3.1154 | 2.9878 | 0.17 | **5.68** |
|  |  |  |  |  |  |  |  | Mass precision (PPM) | 5.76 | 4.96 | 4.65 | 5.12 |  |  |
|  |  |  |  |  |  |  |  | Index of peak quality (IPQ) | 0.03 | 0.04 | 0.04 | 0.04 |  |  |
|  |  |  |  |  |  |  |  | Signal-to-noise ratio (S/N) | 111.70 | 178.68 | 115.26 | 135.21 |  |  |
| AH | 3 | 4 | 1 | 0 | 1851.9562 | | 1851.9448 | Relative % area | 1.9289 | 1.7457 | 1.7878 | 1.8208 | 0.08 | **4.30** |
|  |  |  |  |  |  |  |  | Mass precision (PPM) | 6.83 | 5.68 | 5.91 | 6.14 |  |  |
|  |  |  |  |  |  |  |  | Index of peak quality (IPQ) | 0.04 | 0.04 | 0.05 | 0.05 |  |  |
|  |  |  |  |  |  |  |  | Signal-to-noise ratio (S/N) | 204.11 | 266.15 | 124.88 | 198.38 |  |  |
| AI | 4 | 4 | 0 | 0 | 1881.9668 | | 1881.9593 | Relative % area | 7.3665 | 7.2162 | 6.9749 | 7.1859 | 0.16 | **2.24** |
|  |  |  |  |  |  |  |  | Mass precision (PPM) | 4.83 | 3.39 | 3.67 | 3.96 |  |  |
|  |  |  |  |  |  |  |  | Index of peak quality (IPQ) | 0.15 | 0.16 | 0.15 | 0.15 |  |  |
|  |  |  |  |  |  |  |  | Signal-to-noise ratio (S/N) | 1098.60 | 1018.75 | 575.87 | 897.74 |  |  |
| AJ | 3 | 2 | 2 | 1 | 1896.9664 | | 1896.9544 | Relative % area | 0.5973 | 0.6764 | 0.6495 | 0.6410 | 0.03 | **5.13** |
|  |  |  |  |  |  |  |  | Mass precision (PPM) | 7.14 | 6.10 | 5.77 | 6.34 |  |  |
|  |  |  |  |  |  |  |  | Index of peak quality (IPQ) | 0.14 | 0.14 | 0.20 | 0.16 |  |  |
|  |  |  |  |  |  |  |  | Signal-to-noise ratio (S/N) | 59.22 | 86.73 | 27.57 | 57.84 |  |  |
| AK | 3 | 3 | 1 | 1 | 1968.0036 | | 1967.9914 | Relative % area | 5.0337 | 4.7588 | 4.4678 | 4.7534 | 0.23 | **4.86** |
|  |  |  |  |  |  |  |  | Mass precision (PPM) | 6.99 | 5.71 | 5.78 | 6.16 |  |  |
|  |  |  |  |  |  |  |  | Index of peak quality (IPQ) | 0.01 | 0.03 | 0.03 | 0.03 |  |  |
|  |  |  |  |  |  |  |  | Signal-to-noise ratio (S/N) | 487.93 | 539.16 | 310.40 | 445.83 |  |  |
| AL | 2 | 4 | 1 | 1 | 2009.0301 | | 2009.0147 | Relative % area | 0.4783 | 0.4549 | 0.2581 | 0.3971 | 0.10 | **24.86** |
|  |  |  |  |  |  |  |  | Mass precision (PPM) | 8.59 | 6.34 | 7.99 | 7.64 |  |  |
|  |  |  |  |  |  |  |  | Index of peak quality (IPQ) | 0.15 | 0.13 | 0.30 | 0.19 |  |  |
|  |  |  |  |  |  |  |  | Signal-to-noise ratio (S/N) | 30.35 | 52.03 | 23.40 | 35.26 |  |  |
| AM | 3 | 4 | 2 | 0 | 2026.0454 | | 2026.0317 | Relative % area | 0.5492 | 0.4194 | 0.4257 | 0.4648 | 0.06 | **12.86** |
|  |  |  |  |  |  |  |  | Mass precision (PPM) | 7.19 | 6.21 | 6.94 | 6.78 |  |  |
|  |  |  |  |  |  |  |  | Index of peak quality (IPQ) | 0.19 | 0.15 | 0.27 | 0.20 |  |  |
|  |  |  |  |  |  |  |  | Signal-to-noise ratio (S/N) | 44.95 | 42.77 | 18.92 | 35.55 |  |  |
| AN | 4 | 4 | 1 | 0 | 2056.0560 | | 2056.0412 | Relative % area | 3.8373 | 3.2588 | 3.1715 | 3.4225 | 0.30 | **8.63** |
|  |  |  |  |  |  |  |  | Mass precision (PPM) | 8.11 | 6.73 | 6.66 | 7.17 |  |  |
|  |  |  |  |  |  |  |  | Index of peak quality (IPQ) | 0.05 | 0.03 | 0.04 | 0.04 |  |  |
|  |  |  |  |  |  |  |  | Signal-to-noise ratio (S/N) | 353.38 | 421.67 | 173.07 | 316.04 |  |  |
| AO | 3 | 3 | 2 | 1 | 2142.0928 | | 2142.0760 | Relative % area | 1.4257 | 1.2416 | 1.2208 | 1.2960 | 0.09 | **7.11** |
|  |  |  |  |  |  |  |  | Mass precision (PPM) | 8.96 | 7.30 | 7.28 | 7.85 |  |  |
|  |  |  |  |  |  |  |  | Index of peak quality (IPQ) | 0.10 | 0.13 | 0.05 | 0.09 |  |  |
|  |  |  |  |  |  |  |  | Signal-to-noise ratio (S/N) | 122.42 | 121.26 | 66.83 | 103.50 |  |  |
|  |  |  |  |  |  | |  |  |  |  |  |  |  |  |
|  |  |  |  |  |  | |  |  |  |  |  |  |  |  |
| AP | 3 | 3 | 0 | 2 | 2155.0880 | | 2155.0711 | Relative % area | 0.6771 | 0.5894 | 0.7680 | 0.6782 | 0.07 | **10.75** |
|  |  |  |  |  |  |  |  | Mass precision (PPM) | 8.96 | 7.48 | 7.08 | 7.84 |  |  |
|  |  |  |  |  |  |  |  | Index of peak quality (IPQ) | 0.11 | 0.07 | 0.21 | 0.13 |  |  |
|  |  |  |  |  |  |  |  | Signal-to-noise ratio (S/N) | 58.20 | 75.32 | 34.21 | 55.91 |  |  |
| AQ | 3 | 4 | 1 | 1 | 2213.1299 | | 2213.1111 | Relative % area | 1.6490 | 1.4323 | 1.4044 | 1.4952 | 0.11 | **7.31** |
|  |  |  |  |  |  |  |  | Mass precision (PPM) | 9.61 | 7.88 | 7.91 | 8.47 |  |  |
|  |  |  |  |  |  |  |  | Index of peak quality (IPQ) | 0.23 | 0.23 | 0.21 | 0.23 |  |  |
|  |  |  |  |  |  |  |  | Signal-to-noise ratio (S/N) | 123.41 | 175.56 | 67.32 | 122.10 |  |  |
| AR | 4 | 4 | 2 | 0 | 2230.1452 | | 2230.1277 | Relative % area | 1.3455 | 1.1854 | 1.2364 | 1.2558 | 0.07 | **5.32** |
|  |  |  |  |  |  |  |  | Mass precision (PPM) | 9.19 | 7.35 | 6.93 | 7.82 |  |  |
|  |  |  |  |  |  |  |  | Index of peak quality (IPQ) | 0.06 | 0.05 | 0.16 | 0.09 |  |  |
|  |  |  |  |  |  |  |  | Signal-to-noise ratio (S/N) | 99.03 | 149.43 | 49.35 | 99.27 |  |  |
| AS | 4 | 4 | 0 | 1 | 2243.1404 | | 2243.1212 | Relative % area | 1.4171 | 1.2382 | 1.6283 | 1.4279 | 0.16 | **11.17** |
|  |  |  |  |  |  |  |  | Mass precision (PPM) | 9.76 | 7.93 | 8.03 | 8.57 |  |  |
|  |  |  |  |  |  |  |  | Index of peak quality (IPQ) | 0.05 | 0.04 | 0.07 | 0.06 |  |  |
|  |  |  |  |  |  |  |  | Signal-to-noise ratio (S/N) | 138.79 | 137.75 | 66.06 | 114.20 |  |  |
| AT | 3 | 3 | 1 | 2 | 2329.1772 | | 2329.1566 | Relative % area | 1.2281 | 1.2403 | 1.1578 | 1.2087 | 0.04 | **3.01** |
|  |  |  |  |  |  |  |  | Mass precision (PPM) | 9.91 | 8.25 | 8.33 | 8.83 |  |  |
|  |  |  |  |  |  |  |  | Index of peak quality (IPQ) | 0.11 | 0.06 | 0.12 | 0.10 |  |  |
|  |  |  |  |  |  |  |  | Signal-to-noise ratio (S/N) | 92.21 | 167.45 | 39.97 | 99.88 |  |  |

**Table 11.** PaTu S human pancreatic cancer cell line *O*-glycan identification and quantitation. Glycan compositions, calculated *m/z* values, average registered *m/z* values from three sample replicates (Avg. *m/z*), relative areas, mass precision (PPM), index of peak quality (IPQ), signal-to-noise (S/N) ratios, standard deviations (SDs) and coefficients of variation (CVs) for quantified *O*-glycan structures detected in PaTu S human pancreatic cancer cell line and calculated after triplicate analysis. Glycan compositions are given in the terms of hexose (H), *N*-acetylhexosamine (N), deoxyhexose (F), *N*-acetylneuraminic acid (S).

| Peak ID | Composition | | | | *m/z*  (calculated) | Avg *m/z*  (registered) | Criteria | PaTu S *O*-glycan data | | | Average | Variation | |
| --- | --- | --- | --- | --- | --- | --- | --- | --- | --- | --- | --- | --- | --- |
|  | Hex (H) | HexNAc (N) | Fuc (F) | Neu5Ac (S) |  |  |  | Replicate A | Replicate B | Replicate C |  | SD | **CV** |
| A | 0 | 2 | 0 | 0 | 575.3150 | 575.3129 | Relative % area | 0.8504 | 0.7237 | 0.8763 | 0.8168 | 0.07 | **8.16** |
|  |  |  |  |  |  |  | Mass precision (PPM) | 3.56 | 4.14 | 3.39 | 3.70 |  |  |
|  |  |  |  |  |  |  | Index of peak quality (IPQ) | 0.31 | 0.14 | 0.02 | 0.15 |  |  |
|  |  |  |  |  |  |  | Signal-to-noise ratio (S/N) | 103.02 | 141.21 | 85.60 | 109.94 |  |  |
| B | 0 | 1 | 0 | 1 | 691.3624 | 691.3613 | Relative % area | 0.7776 | 1.0614 | 1.3799 | 1.0729 | 0.25 | **22.93** |
|  |  |  |  |  |  |  | Mass precision (PPM) | 1.70 | 1.57 | 1.57 | 1.61 |  |  |
|  |  |  |  |  |  |  | Index of peak quality (IPQ) | 0.19 | 0.10 | 0.21 | 0.17 |  |  |
|  |  |  |  |  |  |  | Signal-to-noise ratio (S/N) | 104.57 | 141.09 | 52.03 | 99.23 |  |  |
| C | 2 | 1 | 0 | 0 | 738.3883 | 738.3873 | Relative % area | 0.7232 | 0.8796 | 1.1947 | 0.9325 | 0.20 | **21.03** |
|  |  |  |  |  |  |  | Mass precision (PPM) | 1.55 | 1.40 | 0.90 | 1.29 |  |  |
|  |  |  |  |  |  |  | Index of peak quality (IPQ) | 0.06 | 0.07 | 0.11 | 0.08 |  |  |
|  |  |  |  |  |  |  | Signal-to-noise ratio (S/N) | 324.57 | 215.41 | 146.95 | 228.98 |  |  |
| D | 1 | 2 | 0 | 0 | 779.4148 | 779.4144 | Relative % area | 1.4301 | 1.3156 | 2.7019 | 1.8159 | 0.63 | **34.60** |
|  |  |  |  |  |  |  | Mass precision (PPM) | 0.57 | 0.39 | 0.49 | 0.48 |  |  |
|  |  |  |  |  |  |  | Index of peak quality (IPQ) | 0.09 | 0.04 | 0.07 | 0.07 |  |  |
|  |  |  |  |  |  |  | Signal-to-noise ratio (S/N) | 508.89 | 274.12 | 287.72 | 356.91 |  |  |
| E | 1 | 1 | 0 | 1 | 895.4621 | 895.4617 | Relative % area | 1.7318 | 2.1047 | 3.4153 | 2.4173 | 0.72 | **29.87** |
|  |  |  |  |  |  |  | Mass precision (PPM) | 0.59 | 0.77 | 0.15 | 0.51 |  |  |
|  |  |  |  |  |  |  | Index of peak quality (IPQ) | 0.06 | 0.05 | 0.08 | 0.06 |  |  |
|  |  |  |  |  |  |  | Signal-to-noise ratio (S/N) | 134.06 | 380.91 | 289.35 | 268.11 |  |  |
| F | 2 | 2 | 0 | 0 | 983.5146 | 983.5144 | Relative % area | 7.5804 | 7.3548 | 9.0254 | 7.9868 | 0.74 | **9.27** |
|  |  |  |  |  |  |  | Mass precision (PPM) | 0.09 | 0.13 | 0.41 | 0.21 |  |  |
|  |  |  |  |  |  |  | Index of peak quality (IPQ) | 0.04 | 0.05 | 0.04 | 0.04 |  |  |
|  |  |  |  |  |  |  | Signal-to-noise ratio (S/N) | 2181.86 | 1993.82 | 1326.23 | 1833.97 |  |  |
| G | 1 | 3 | 0 | 0 | 1024.5411 | 1024.5401 | Relative % area | 0.5074 | 0.4691 | 0.5324 | 0.5030 | 0.03 | **5.18** |
|  |  |  |  |  |  |  | Mass precision (PPM) | 1.09 | 1.10 | 0.96 | 1.05 |  |  |
|  |  |  |  |  |  |  | Index of peak quality (IPQ) | 0.08 | 0.03 | 0.15 | 0.09 |  |  |
|  |  |  |  |  |  |  | Signal-to-noise ratio (S/N) | 140.39 | 97.82 | 78.72 | 105.64 |  |  |
| H | 1 | 2 | 0 | 1 | 1140.5885 | 1140.5875 | Relative % area | 0.6404 | 0.8108 | 0.7808 | 0.7440 | 0.07 | **9.98** |
|  |  |  |  |  |  |  | Mass precision (PPM) | 0.90 | 1.09 | 0.65 | 0.88 |  |  |
|  |  |  |  |  |  |  | Index of peak quality (IPQ) | 0.09 | 0.09 | 0.06 | 0.08 |  |  |
|  |  |  |  |  |  |  | Signal-to-noise ratio (S/N) | 100.59 | 126.06 | 59.26 | 95.30 |  |  |
| I | 2 | 2 | 1 | 0 | 1157.6038 | 1157.6036 | Relative % area | 1.5168 | 1.6981 | 2.0306 | 1.7485 | 0.21 | **12.17** |
|  |  |  |  |  |  |  | Mass precision (PPM) | 0.17 | 0.04 | 0.25 | 0.15 |  |  |
|  |  |  |  |  |  |  | Index of peak quality (IPQ) | 0.18 | 0.17 | 0.20 | 0.18 |  |  |
|  |  |  |  |  |  |  | Signal-to-noise ratio (S/N) | 245.07 | 294.38 | 206.71 | 248.72 |  |  |
| J | 3 | 2 | 0 | 0 | 1187.6144 | 1187.6133 | Relative % area | 3.6993 | 3.7059 | 3.6987 | 3.7013 | 0.00 | **0.09** |
|  |  |  |  |  |  |  | Mass precision (PPM) | 0.89 | 1.15 | 0.61 | 0.88 |  |  |
|  |  |  |  |  |  |  | Index of peak quality (IPQ) | 0.02 | 0.04 | 0.01 | 0.02 |  |  |
|  |  |  |  |  |  |  | Signal-to-noise ratio (S/N) | 716.03 | 768.15 | 456.61 | 646.93 |  |  |
| K | 1 | 3 | 1 | 0 | 1198.6303 | 1198.6298 | Relative % area | 0.3932 | 0.4798 | 0.4096 | 0.4275 | 0.04 | **8.79** |
|  |  |  |  |  |  |  | Mass precision (PPM) | 0.42 | 0.03 | 0.77 | 0.41 |  |  |
|  |  |  |  |  |  |  | Index of peak quality (IPQ) | 0.13 | 0.04 | 0.15 | 0.11 |  |  |
|  |  |  |  |  |  |  | Signal-to-noise ratio (S/N) | 52.08 | 74.90 | 52.69 | 59.89 |  |  |
| L | 2 | 3 | 0 | 0 | 1228.6409 | 1228.6397 | Relative % area | 1.8147 | 1.8745 | 1.8016 | 1.8302 | 0.03 | **1.73** |
|  |  |  |  |  |  |  | Mass precision (PPM) | 0.88 | 1.09 | 0.86 | 0.94 |  |  |
|  |  |  |  |  |  |  | Index of peak quality (IPQ) | 0.04 | 0.03 | 0.10 | 0.06 |  |  |
|  |  |  |  |  |  |  | Signal-to-noise ratio (S/N) | 237.17 | 253.05 | 188.81 | 226.34 |  |  |
| M | 1 | 1 | 0 | 2 | 1256.6358 | 1256.6342 | Relative % area | 2.3163 | 2.7618 | 3.0896 | 2.7226 | 0.32 | **11.64** |
|  |  |  |  |  |  |  | Mass precision (PPM) | 1.17 | 1.52 | 1.11 | 1.27 |  |  |
|  |  |  |  |  |  |  | Index of peak quality (IPQ) | 0.03 | 0.05 | 0.07 | 0.05 |  |  |
|  |  |  |  |  |  |  | Signal-to-noise ratio (S/N) | 404.38 | 429.21 | 233.66 | 355.75 |  |  |
| N | 1 | 4 | 0 | 0 | 1269.6675 | 1269.6669 | Relative % area | 0.6903 | 0.6293 | 0.7765 | 0.6987 | 0.06 | **8.64** |
|  |  |  |  |  |  |  | Mass precision (PPM) | 0.56 | 0.32 | 0.49 | 0.46 |  |  |
|  |  |  |  |  |  |  | Index of peak quality (IPQ) | 0.10 | 0.08 | 0.03 | 0.07 |  |  |
|  |  |  |  |  |  |  | Signal-to-noise ratio (S/N) | 94.43 | 122.25 | 60.31 | 92.33 |  |  |
| O | 2 | 2 | 0 | 1 | 1344.6882 | 1344.6869 | Relative % area | 4.7776 | 5.8042 | 5.9546 | 5.5121 | 0.52 | **9.49** |
|  |  |  |  |  |  |  | Mass precision (PPM) | 0.86 | 1.36 | 0.68 | 0.97 |  |  |
|  |  |  |  |  |  |  | Index of peak quality (IPQ) | 0.07 | 0.05 | 0.06 | 0.06 |  |  |
|  |  |  |  |  |  |  | Signal-to-noise ratio (S/N) | 580.01 | 709.86 | 515.96 | 601.94 |  |  |
| P | 2 | 3 | 1 | 0 | 1402.7301 | 1402.7285 | Relative % area | 2.6441 | 2.8086 | 3.2258 | 2.8928 | 0.24 | **8.46** |
|  |  |  |  |  |  |  | Mass precision (PPM) | 1.01 | 1.60 | 0.79 | 1.14 |  |  |
|  |  |  |  |  |  |  | Index of peak quality (IPQ) | 0.04 | 0.07 | 0.02 | 0.04 |  |  |
|  |  |  |  |  |  |  | Signal-to-noise ratio (S/N) | 234.76 | 284.29 | 183.17 | 234.07 |  |  |
| Q | 3 | 3 | 0 | 0 | 1432.7407 | 1432.7392 | Relative % area | 5.8420 | 5.7423 | 5.1040 | 5.5628 | 0.33 | **5.88** |
|  |  |  |  |  |  |  | Mass precision (PPM) | 1.08 | 1.32 | 0.77 | 1.06 |  |  |
|  |  |  |  |  |  |  | Index of peak quality (IPQ) | 0.02 | 0.04 | 0.07 | 0.04 |  |  |
|  |  |  |  |  |  |  | Signal-to-noise ratio (S/N) | 504.07 | 700.41 | 332.77 | 512.42 |  |  |
| R | 2 | 4 | 0 | 0 | 1473.7672 | 1473.7663 | Relative % area | 3.1702 | 3.1267 | 3.0802 | 3.1257 | 0.04 | **1.18** |
|  |  |  |  |  |  |  | Mass precision (PPM) | 0.69 | 0.85 | 0.33 | 0.63 |  |  |
|  |  |  |  |  |  |  | Index of peak quality (IPQ) | 0.05 | 0.04 | 0.03 | 0.04 |  |  |
|  |  |  |  |  |  |  | Signal-to-noise ratio (S/N) | 333.31 | 362.06 | 199.57 | 298.31 |  |  |
| S | 2 | 2 | 1 | 1 | 1518.7775 | 1518.7770 | Relative % area | 1.9784 | 1.7694 | 1.9462 | 1.8980 | 0.09 | **4.84** |
|  |  |  |  |  |  |  | Mass precision (PPM) | 0.05 | 0.57 | 0.32 | 0.31 |  |  |
|  |  |  |  |  |  |  | Index of peak quality (IPQ) | 0.06 | 0.17 | 0.09 | 0.11 |  |  |
|  |  |  |  |  |  |  | Signal-to-noise ratio (S/N) | 82.14 | 71.17 | 68.32 | 73.88 |  |  |
|  |  |  |  |  |  |  |  |  |  |  |  |  |  |
| T | 2 | 3 | 2 | 0 | 1576.8193 | 1576.8189 | Relative % area | 0.8362 | 0.9659 | 1.2249 | 1.0090 | 0.16 | **16.01** |
|  |  |  |  |  |  |  | Mass precision (PPM) | 0.21 | 0.41 | 0.13 | 0.25 |  |  |
|  |  |  |  |  |  |  | Index of peak quality (IPQ) | 0.13 | 0.14 | 0.22 | 0.16 |  |  |
|  |  |  |  |  |  |  | Signal-to-noise ratio (S/N) | 63.81 | 75.45 | 48.35 | 62.54 |  |  |
| U | 3 | 3 | 1 | 0 | 1606.8299 | 1606.8291 | Relative % area | 2.7208 | 2.7758 | 2.8786 | 2.7917 | 0.07 | **2.34** |
|  |  |  |  |  |  |  | Mass precision (PPM) | 0.52 | 0.71 | 0.21 | 0.48 |  |  |
|  |  |  |  |  |  |  | Index of peak quality (IPQ) | 0.08 | 0.08 | 0.12 | 0.09 |  |  |
|  |  |  |  |  |  |  | Signal-to-noise ratio (S/N) | 130.13 | 157.36 | 82.77 | 123.42 |  |  |
| V | 4 | 3 | 0 | 0 | 1636.8404 | 1636.8392 | Relative % area | 0.9978 | 0.8739 | 0.6942 | 0.8553 | 0.12 | **14.57** |
|  |  |  |  |  |  |  | Mass precision (PPM) | 0.42 | 1.09 | 0.81 | 0.77 |  |  |
|  |  |  |  |  |  |  | Index of peak quality (IPQ) | 0.04 | 0.13 | 0.04 | 0.07 |  |  |
|  |  |  |  |  |  |  | Signal-to-noise ratio (S/N) | 68.96 | 51.63 | 25.25 | 48.61 |  |  |
| W | 2 | 4 | 1 | 0 | 1647.8564 | 1647.8561 | Relative % area | 0.9175 | 0.8163 | 0.7528 | 0.8289 | 0.07 | **8.19** |
|  |  |  |  |  |  |  | Mass precision (PPM) | 0.05 | 0.00 | 0.46 | 0.17 |  |  |
|  |  |  |  |  |  |  | Index of peak quality (IPQ) | 0.19 | 0.20 | 0.30 | 0.23 |  |  |
|  |  |  |  |  |  |  | Signal-to-noise ratio (S/N) | 45.96 | 66.67 | 39.72 | 50.78 |  |  |
| X | 3 | 4 | 0 | 0 | 1677.8670 | 1677.8666 | Relative % area | 9.8691 | 9.6208 | 9.0123 | 9.5007 | 0.36 | **3.79** |
|  |  |  |  |  |  |  | Mass precision (PPM) | 0.21 | 0.29 | 0.15 | 0.22 |  |  |
|  |  |  |  |  |  |  | Index of peak quality (IPQ) | 0.04 | 0.02 | 0.05 | 0.04 |  |  |
|  |  |  |  |  |  |  | Signal-to-noise ratio (S/N) | 511.70 | 855.75 | 294.48 | 553.98 |  |  |
| Y | 2 | 2 | 0 | 2 | 1705.8619 | 1705.8615 | Relative % area | 1.6359 | 2.1422 | 1.5737 | 1.7839 | 0.25 | **14.27** |
|  |  |  |  |  |  |  | Mass precision (PPM) | 0.14 | 0.20 | 0.38 | 0.24 |  |  |
|  |  |  |  |  |  |  | Index of peak quality (IPQ) | 0.08 | 0.09 | 0.09 | 0.09 |  |  |
|  |  |  |  |  |  |  | Signal-to-noise ratio (S/N) | 95.15 | 152.89 | 63.60 | 103.88 |  |  |
| Z | 2 | 5 | 0 | 0 | 1718.8935 | 1718.8894 | Relative % area | 0.3701 | 0.3630 | 0.4403 | 0.3911 | 0.03 | **8.92** |
|  |  |  |  |  |  |  | Mass precision (PPM) | 2.08 | 2.61 | 2.55 | 2.41 |  |  |
|  |  |  |  |  |  |  | Index of peak quality (IPQ) | 0.07 | 0.23 | 0.20 | 0.17 |  |  |
|  |  |  |  |  |  |  | Signal-to-noise ratio (S/N) | 26.27 | 35.75 | 18.83 | 26.95 |  |  |
| AA | 2 | 3 | 1 | 1 | 1763.9038 | 1763.8962 | Relative % area | 1.2277 | 0.9068 | 1.3690 | 1.1678 | 0.19 | **16.56** |
|  |  |  |  |  |  |  | Mass precision (PPM) | 7.48 | 1.09 | 4.40 | 4.32 |  |  |
|  |  |  |  |  |  |  | Index of peak quality (IPQ) | 0.20 | 0.07 | 0.06 | 0.11 |  |  |
|  |  |  |  |  |  |  | Signal-to-noise ratio (S/N) | 41.36 | 74.86 | 39.61 | 51.94 |  |  |
| AB | 3 | 3 | 0 | 1 | 1793.9143 | 1793.9134 | Relative % area | 1.4896 | 1.6915 | 1.4791 | 1.5534 | 0.10 | **6.29** |
|  |  |  |  |  |  |  | Mass precision (PPM) | 0.36 | 0.04 | 1.18 | 0.53 |  |  |
|  |  |  |  |  |  |  | Index of peak quality (IPQ) | 0.05 | 0.04 | 0.09 | 0.06 |  |  |
|  |  |  |  |  |  |  | Signal-to-noise ratio (S/N) | 75.18 | 138.70 | 52.89 | 88.92 |  |  |
| AC | 3 | 4 | 1 | 0 | 1851.9562 | 1851.9541 | Relative % area | 2.5151 | 2.3960 | 1.9271 | 2.2794 | 0.25 | **11.14** |
|  |  |  |  |  |  |  | Mass precision (PPM) | 1.16 | 0.44 | 1.84 | 1.14 |  |  |
|  |  |  |  |  |  |  | Index of peak quality (IPQ) | 0.14 | 0.13 | 0.14 | 0.14 |  |  |
|  |  |  |  |  |  |  | Signal-to-noise ratio (S/N) | 98.39 | 148.45 | 60.15 | 102.33 |  |  |
| AD | 4 | 4 | 0 | 0 | 1881.9668 | 1881.9658 | Relative % area | 17.6791 | 16.6288 | 15.0122 | 16.4400 | 1.10 | **6.67** |
|  |  |  |  |  |  |  | Mass precision (PPM) | 0.40 | 0.09 | 1.12 | 0.54 |  |  |
|  |  |  |  |  |  |  | Index of peak quality (IPQ) | 0.04 | 0.03 | 0.04 | 0.04 |  |  |
|  |  |  |  |  |  |  | Signal-to-noise ratio (S/N) | 736.30 | 1106.48 | 482.62 | 775.13 |  |  |
|  |  |  |  |  |  |  |  |  |  |  |  |  |  |
|  |  |  |  |  |  |  |  |  |  |  |  |  |  |
| AE | 3 | 5 | 0 | 0 | 1922.9933 | 1922.9903 | Relative % area | 1.9268 | 1.7933 | 1.7015 | 1.8072 | 0.09 | **5.12** |
|  |  |  |  |  |  |  | Mass precision (PPM) | 1.23 | 1.46 | 2.02 | 1.57 |  |  |
|  |  |  |  |  |  |  | Index of peak quality (IPQ) | 0.10 | 0.03 | 0.03 | 0.05 |  |  |
|  |  |  |  |  |  |  | Signal-to-noise ratio (S/N) | 113.82 | 127.35 | 59.56 | 100.24 |  |  |
| AF | 4 | 4 | 1 | 0 | 2056.0560 | 2056.0527 | Relative % area | 3.0184 | 2.7875 | 2.4628 | 2.7562 | 0.23 | **8.27** |
|  |  |  |  |  |  |  | Mass precision (PPM) | 1.26 | 1.17 | 2.41 | 1.61 |  |  |
|  |  |  |  |  |  |  | Index of peak quality (IPQ) | 0.27 | 0.27 | 0.17 | 0.24 |  |  |
|  |  |  |  |  |  |  | Signal-to-noise ratio (S/N) | 102.25 | 151.29 | 62.51 | 105.35 |  |  |
| AG | 3 | 5 | 1 | 0 | 2097.0825 | 2097.0785 | Relative % area | 1.2651 | 1.2944 | 1.1489 | 1.2361 | 0.06 | **5.09** |
|  |  |  |  |  |  |  | Mass precision (PPM) | 1.87 | 0.56 | 3.27 | 1.90 |  |  |
|  |  |  |  |  |  |  | Index of peak quality (IPQ) | 0.09 | 0.09 | 0.30 | 0.16 |  |  |
|  |  |  |  |  |  |  | Signal-to-noise ratio (S/N) | 65.08 | 65.22 | 30.09 | 53.46 |  |  |
| AH | 4 | 5 | 0 | 0 | 2127.0931 | 2127.0899 | Relative % area | 2.2363 | 2.1302 | 1.5800 | 1.9822 | 0.29 | **14.51** |
|  |  |  |  |  |  |  | Mass precision (PPM) | 1.62 | 0.98 | 1.95 | 1.51 |  |  |
|  |  |  |  |  |  |  | Index of peak quality (IPQ) | 0.09 | 0.02 | 0.13 | 0.08 |  |  |
|  |  |  |  |  |  |  | Signal-to-noise ratio (S/N) | 86.33 | 131.61 | 38.08 | 85.34 |  |  |
| AI | 4 | 4 | 2 | 0 | 2230.1452 | 2230.1409 | Relative % area | 0.8489 | 0.8737 | 0.7483 | 0.8237 | 0.05 | **6.58** |
|  |  |  |  |  |  |  | Mass precision (PPM) | 2.01 | 1.65 | 2.14 | 1.93 |  |  |
|  |  |  |  |  |  |  | Index of peak quality (IPQ) | 0.23 | 0.27 | 0.17 | 0.22 |  |  |
|  |  |  |  |  |  |  | Signal-to-noise ratio (S/N) | 30.52 | 40.57 | 13.63 | 28.24 |  |  |
| AJ | 4 | 4 | 0 | 1 | 2243.1404 | 2243.1365 | Relative % area | 3.0865 | 2.9691 | 2.1546 | 2.7368 | 0.41 | **15.14** |
|  |  |  |  |  |  |  | Mass precision (PPM) | 1.82 | 0.57 | 2.91 | 1.76 |  |  |
|  |  |  |  |  |  |  | Index of peak quality (IPQ) | 0.04 | 0.06 | 0.18 | 0.09 |  |  |
|  |  |  |  |  |  |  | Signal-to-noise ratio (S/N) | 96.60 | 143.69 | 47.23 | 95.84 |  |  |
| AK | 4 | 5 | 1 | 0 | 2301.1823 | 2301.1761 | Relative % area | 1.8432 | 1.5861 | 1.2278 | 1.5523 | 0.25 | **16.26** |
|  |  |  |  |  |  |  | Mass precision (PPM) | 2.12 | 1.49 | 4.47 | 2.69 |  |  |
|  |  |  |  |  |  |  | Index of peak quality (IPQ) | 0.09 | 0.02 | 0.13 | 0.08 |  |  |
|  |  |  |  |  |  |  | Signal-to-noise ratio (S/N) | 62.37 | 80.45 | 23.47 | 55.43 |  |  |
| AL | 5 | 5 | 0 | 0 | 2331.1929 | 2331.1869 | Relative % area | 1.5830 | 1.4940 | 0.8808 | 1.3192 | 0.31 | **23.66** |
|  |  |  |  |  |  |  | Mass precision (PPM) | 3.03 | 1.21 | 3.40 | 2.55 |  |  |
|  |  |  |  |  |  |  | Index of peak quality (IPQ) | 0.10 | 0.06 | 0.22 | 0.13 |  |  |
|  |  |  |  |  |  |  | Signal-to-noise ratio (S/N) | 60.11 | 71.93 | 20.87 | 50.97 |  |  |
| AM | 4 | 6 | 0 | 0 | 2372.2194 | 2372.2121 | Relative % area | 0.8488 | 0.7705 | 0.7073 | 0.7755 | 0.06 | **7.46** |
|  |  |  |  |  |  |  | Mass precision (PPM) | 1.11 | 1.17 | 6.96 | 3.08 |  |  |
|  |  |  |  |  |  |  | Index of peak quality (IPQ) | 0.21 | 0.07 | 0.36 | 0.22 |  |  |
|  |  |  |  |  |  |  | Signal-to-noise ratio (S/N) | 26.02 | 34.16 | 10.94 | 23.71 |  |  |

**Table 12.** PaTu T human pancreatic cancer cell line *O*-glycan identification and quantitation. Glycan compositions, calculated *m/z* values, average registered *m/z* values from three sample replicates (Avg. *m/z*), relative areas, mass precision (PPM), index of peak quality (IPQ), signal-to-noise (S/N) ratios, standard deviations (SDs) and coefficients of variation (CVs) for quantified *O*-glycan structures detected in PaTu T human pancreatic cancer cell line and calculated after triplicate analysis. Glycan compositions are given in the terms of hexose (H), *N*-acetylhexosamine (N), deoxyhexose (F), *N*-acetylneuraminic acid (S).

| Peak ID | Composition | | | | *m/z*  (calculated) | Avg *m/z*  (registered) | Criteria | PaTu T *O*-glycan data | | | Average | Variation | |
| --- | --- | --- | --- | --- | --- | --- | --- | --- | --- | --- | --- | --- | --- |
|  | Hex (H) | HexNAc (N) | Fuc (F) | Neu5Ac (S) |  |  |  | Replicate A | Replicate B | Replicate C |  | SD | **CV** |
| A | 0 | 1 | 0 | 0 | 330.1887 | 330.1875 | Relative % area | 0.23 | 0.06 | 0.35 | 0.21 | 0.12 | **55.39** |
|  |  |  |  |  |  |  | Mass precision (PPM) | 3.58 | 3.85 | 3.85 | 3.76 |  |  |
|  |  |  |  |  |  |  | Index of peak quality (IPQ) | 0.05 | 0.23 | 0.03 | 0.10 |  |  |
|  |  |  |  |  |  |  | Signal-to-noise ratio (S/N) | 94.92 | 36.34 | 387.19 | 172.82 |  |  |
| B | 2 | 1 | 0 | 0 | 738.3883 | 738.3871 | Relative % area | 17.71 | 11.84 | 9.46 | 13.00 | 3.46 | **26.64** |
|  |  |  |  |  |  |  | Mass precision (PPM) | 1.66 | 1.51 | 1.51 | 1.56 |  |  |
|  |  |  |  |  |  |  | Index of peak quality (IPQ) | 0.08 | 0.06 | 0.05 | 0.06 |  |  |
|  |  |  |  |  |  |  | Signal-to-noise ratio (S/N) | 645.48 | 659.08 | 653.43 | 652.66 |  |  |
| C | 1 | 1 | 0 | 1 | 895.4621 | 895.4614 | Relative % area | 11.92 | 9.87 | 10.41 | 10.73 | 0.86 | **8.06** |
|  |  |  |  |  |  |  | Mass precision (PPM) | 0.90 | 0.85 | 0.85 | 0.86 |  |  |
|  |  |  |  |  |  |  | Index of peak quality (IPQ) | 0.08 | 0.06 | 0.06 | 0.07 |  |  |
|  |  |  |  |  |  |  | Signal-to-noise ratio (S/N) | 338.15 | 472.02 | 380.94 | 397.04 |  |  |
| D | 2 | 2 | 0 | 0 | 983.5146 | 983.5145 | Relative % area | 6.86 | 4.67 | 3.73 | 5.09 | 1.31 | **25.78** |
|  |  |  |  |  |  |  | Mass precision (PPM) | 0.09 | 0.10 | 0.10 | 0.09 |  |  |
|  |  |  |  |  |  |  | Index of peak quality (IPQ) | 0.04 | 0.07 | 0.04 | 0.05 |  |  |
|  |  |  |  |  |  |  | Signal-to-noise ratio (S/N) | 328.02 | 311.20 | 263.47 | 300.90 |  |  |
| E | 1 | 2 | 0 | 1 | 1140.5885 | 1140.5867 | Relative % area | 0.47 | 0.47 | 0.50 | 0.48 | 0.01 | **2.89** |
|  |  |  |  |  |  |  | Mass precision (PPM) | 1.59 | 1.60 | 1.60 | 1.59 |  |  |
|  |  |  |  |  |  |  | Index of peak quality (IPQ) | 0.17 | 0.11 | 0.03 | 0.10 |  |  |
|  |  |  |  |  |  |  | Signal-to-noise ratio (S/N) | 38.00 | 33.99 | 59.13 | 43.70 |  |  |
| F | 2 | 2 | 1 | 0 | 1157.6038 | 1157.6029 | Relative % area | 0.85 | 0.47 | 0.52 | 0.61 | 0.17 | **27.68** |
|  |  |  |  |  |  |  | Mass precision (PPM) | 1.36 | 0.47 | 0.56 | 0.80 |  |  |
|  |  |  |  |  |  |  | Index of peak quality (IPQ) | 0.10 | 0.17 | 0.32 | 0.20 |  |  |
|  |  |  |  |  |  |  | Signal-to-noise ratio (S/N) | 17.06 | 14.57 | 21.34 | 17.66 |  |  |
| G | 3 | 2 | 0 | 0 | 1187.6144 | 1187.6129 | Relative % area | 2.98 | 2.55 | 1.87 | 2.47 | 0.46 | **18.55** |
|  |  |  |  |  |  |  | Mass precision (PPM) | 1.52 | 1.12 | 1.12 | 1.25 |  |  |
|  |  |  |  |  |  |  | Index of peak quality (IPQ) | 0.12 | 0.22 | 0.14 | 0.16 |  |  |
|  |  |  |  |  |  |  | Signal-to-noise ratio (S/N) | 80.45 | 85.85 | 76.69 | 80.99 |  |  |
| H | 1 | 1 | 0 | 2 | 1256.6358 | 1256.6329 | Relative % area | 27.04 | 30.96 | 34.15 | 30.72 | 2.91 | **9.47** |
|  |  |  |  |  |  |  | Mass precision (PPM) | 2.55 | 2.15 | 2.15 | 2.29 |  |  |
|  |  |  |  |  |  |  | Index of peak quality (IPQ) | 0.05 | 0.04 | 0.03 | 0.04 |  |  |
|  |  |  |  |  |  |  | Signal-to-noise ratio (S/N) | 466.77 | 628.18 | 1079.65 | 724.87 |  |  |
| I | 2 | 2 | 0 | 1 | 1344.6882 | 1344.6844 | Relative % area | 9.65 | 9.13 | 9.24 | 9.34 | 0.22 | **2.40** |
|  |  |  |  |  |  |  | Mass precision (PPM) | 2.80 | 2.71 | 3.00 | 2.84 |  |  |
|  |  |  |  |  |  |  | Index of peak quality (IPQ) | 0.06 | 0.09 | 0.08 | 0.08 |  |  |
|  |  |  |  |  |  |  | Signal-to-noise ratio (S/N) | 202.20 | 248.03 | 346.28 | 265.51 |  |  |
| J | 3 | 2 | 1 | 0 | 1361.7036 | 1361.6997 | Relative % area | 2.27 | 1.69 | 1.64 | 1.87 | 0.28 | **15.24** |
|  |  |  |  |  |  |  | Mass precision (PPM) | 3.00 | 2.61 | 2.90 | 2.84 |  |  |
|  |  |  |  |  |  |  | Index of peak quality (IPQ) | 0.09 | 0.18 | 0.24 | 0.17 |  |  |
|  |  |  |  |  |  |  | Signal-to-noise ratio (S/N) | 36.91 | 52.29 | 56.30 | 48.50 |  |  |
| K | 3 | 3 | 1 | 0 | 1606.8299 | 1606.8229 | Relative % area | 1.75 | 1.15 | 1.48 | 1.46 | 0.25 | **17.01** |
|  |  |  |  |  |  |  | Mass precision (PPM) | 3.68 | 4.78 | 4.53 | 4.33 |  |  |
|  |  |  |  |  |  |  | Index of peak quality (IPQ) | 0.16 | 0.27 | 0.15 | 0.19 |  |  |
|  |  |  |  |  |  |  | Signal-to-noise ratio (S/N) | 12.79 | 21.21 | 20.74 | 18.25 |  |  |
| L | 2 | 2 | 0 | 2 | 1705.8619 | 1705.8546 | Relative % area | 18.28 | 27.13 | 26.65 | 24.02 | 4.06 | **16.92** |
|  |  |  |  |  |  |  | Mass precision (PPM) | 3.76 | 4.55 | 4.55 | 4.29 |  |  |
|  |  |  |  |  |  |  | Index of peak quality (IPQ) | 0.06 | 0.06 | 0.03 | 0.05 |  |  |
|  |  |  |  |  |  |  | Signal-to-noise ratio (S/N) | 114.55 | 247.37 | 301.10 | 221.01 |  |  |


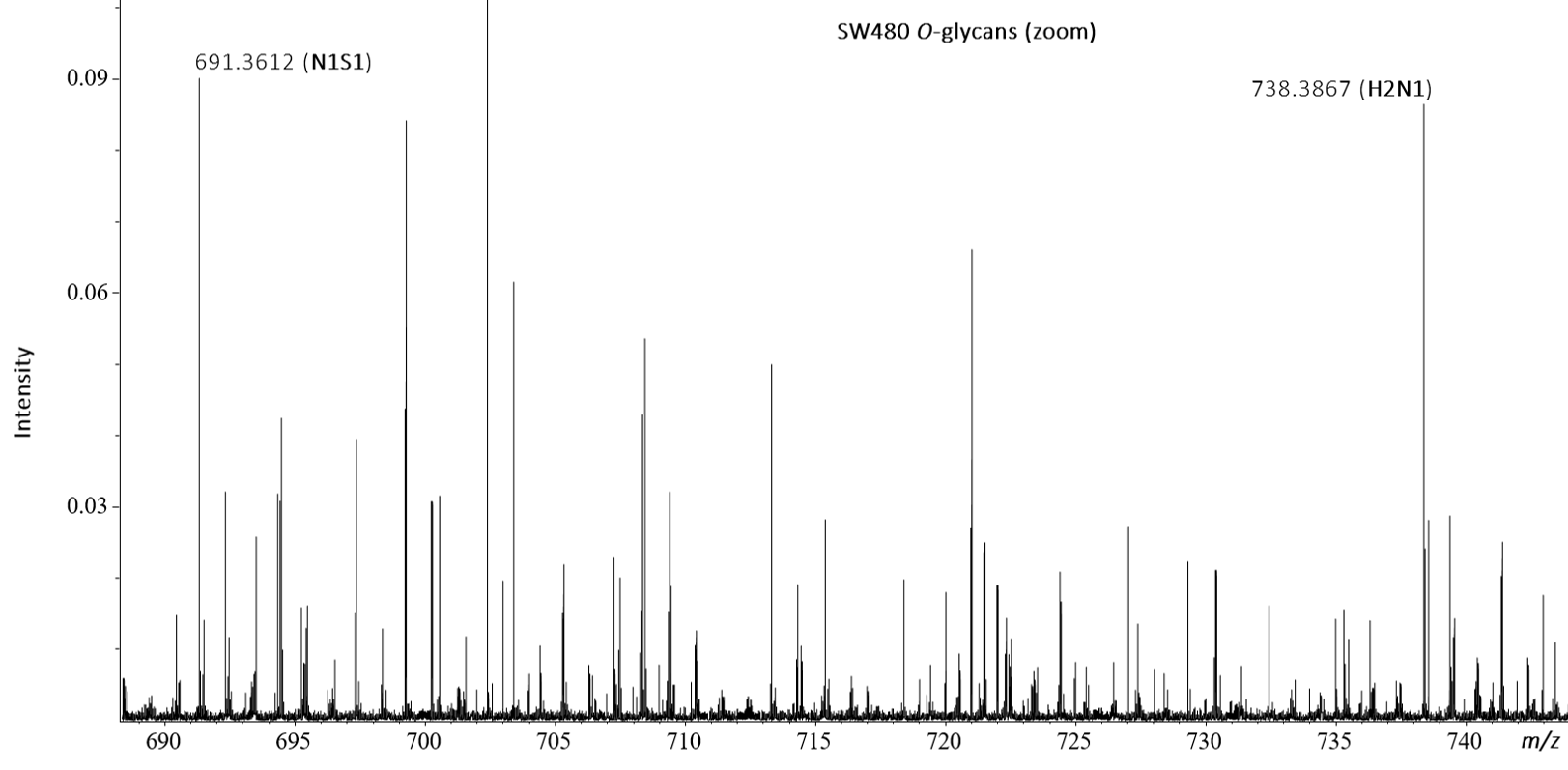


**Fig. 20.** Expanded view of the low mass region of MALDI-FTICR-MS spectra for SW480 human colorectal cancer cell line with annotated m/z values and compositions for detected *O*-glycans. Glycan compositions are given in the terms of hexose (H), N-acetylhexosamine (N), deoxyhexose (F), N-acetylneuraminic acid (S).


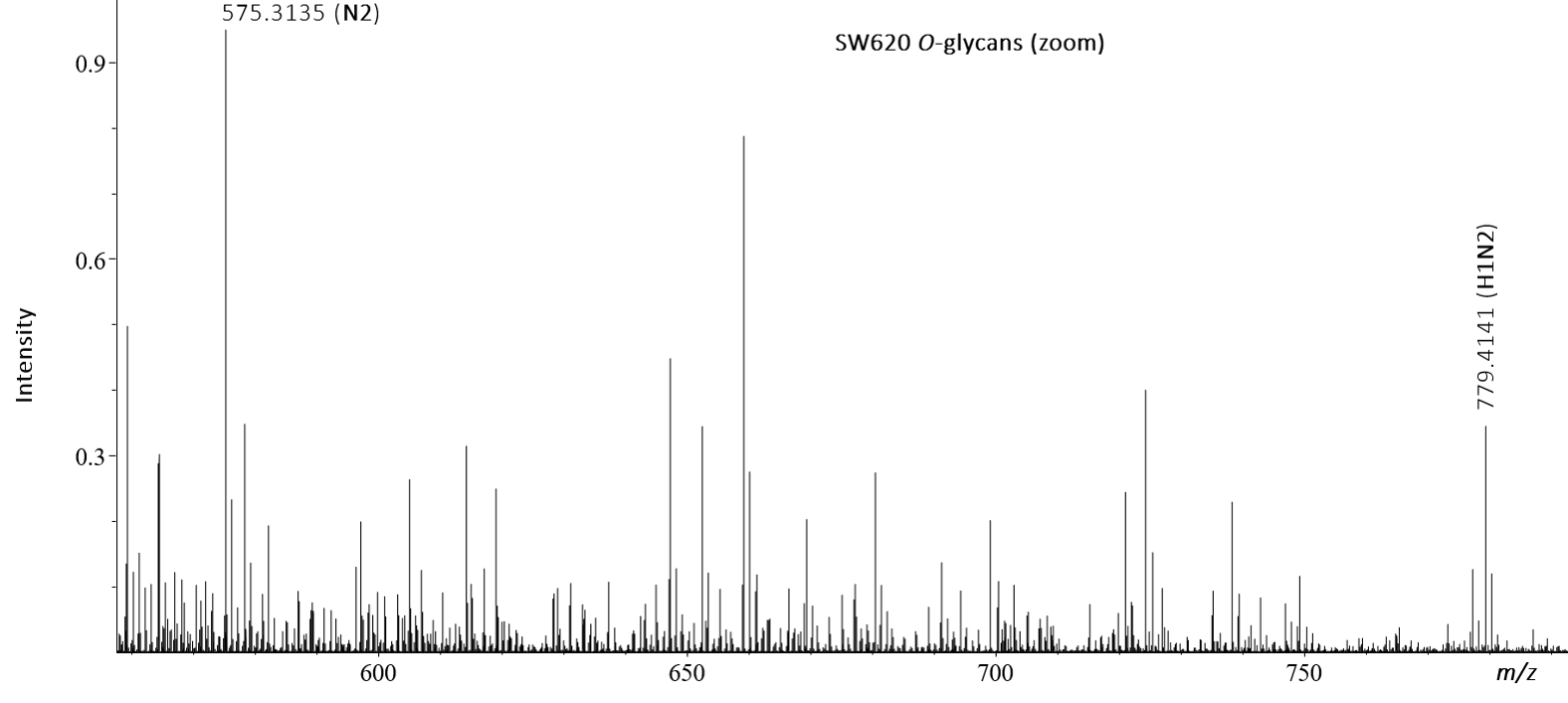


**Fig. 21.** Expanded view of the low mass region of MALDI-FTICR-MS spectra for SW620 human colorectal cancer cell line with annotated m/z values and compositions for detected *O*-glycans. Glycan compositions are given in the terms of hexose (H), N-acetylhexosamine (N), deoxyhexose (F).


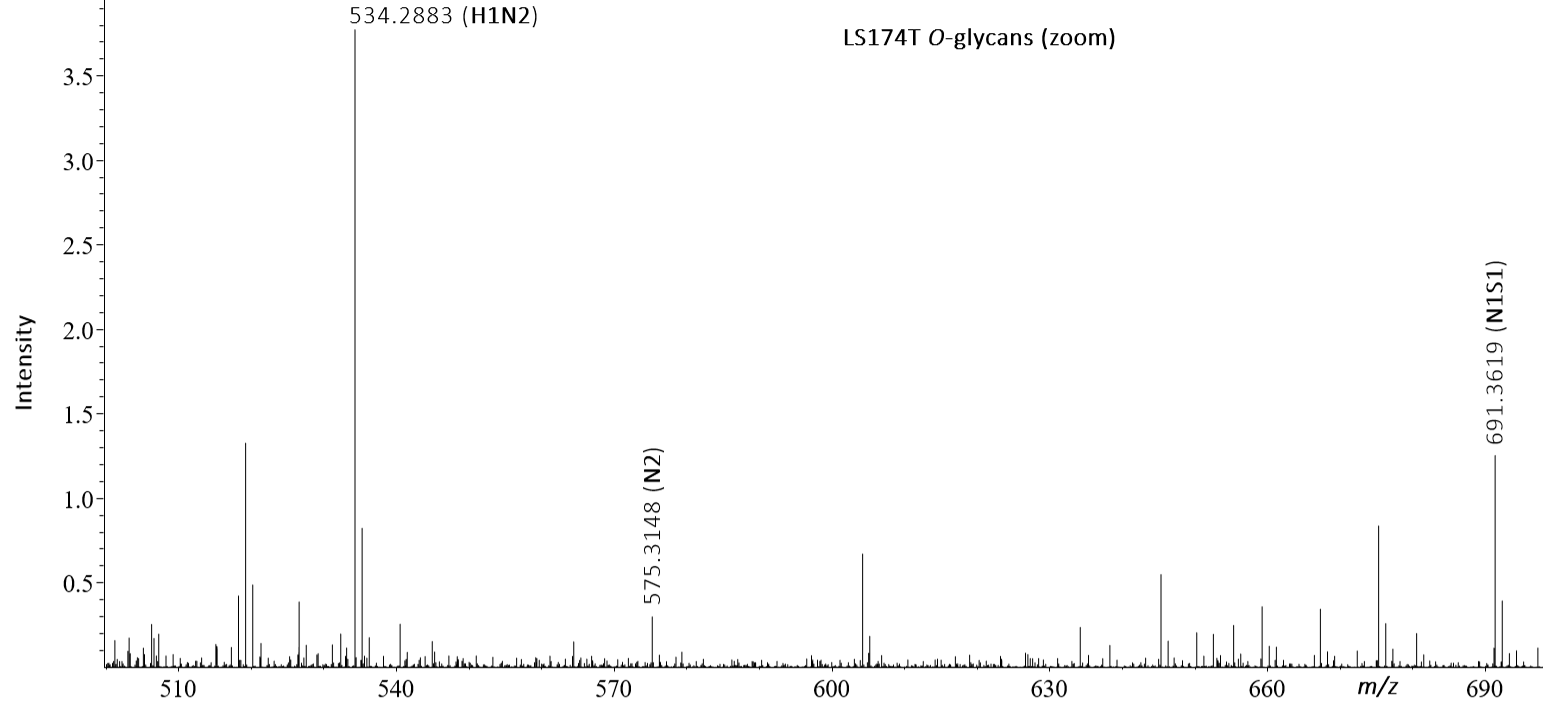


**Fig. 22.** Expanded view of the low mass region of MALDI-FTICR-MS spectra for LS174T human colorectal cancer cell line with annotated m/z values and compositions for detected *O*-glycans. Glycan compositions are given in the terms of hexose (H), N-acetylhexosamine (N), deoxyhexose (F), N-acetylneuraminic acid (S).


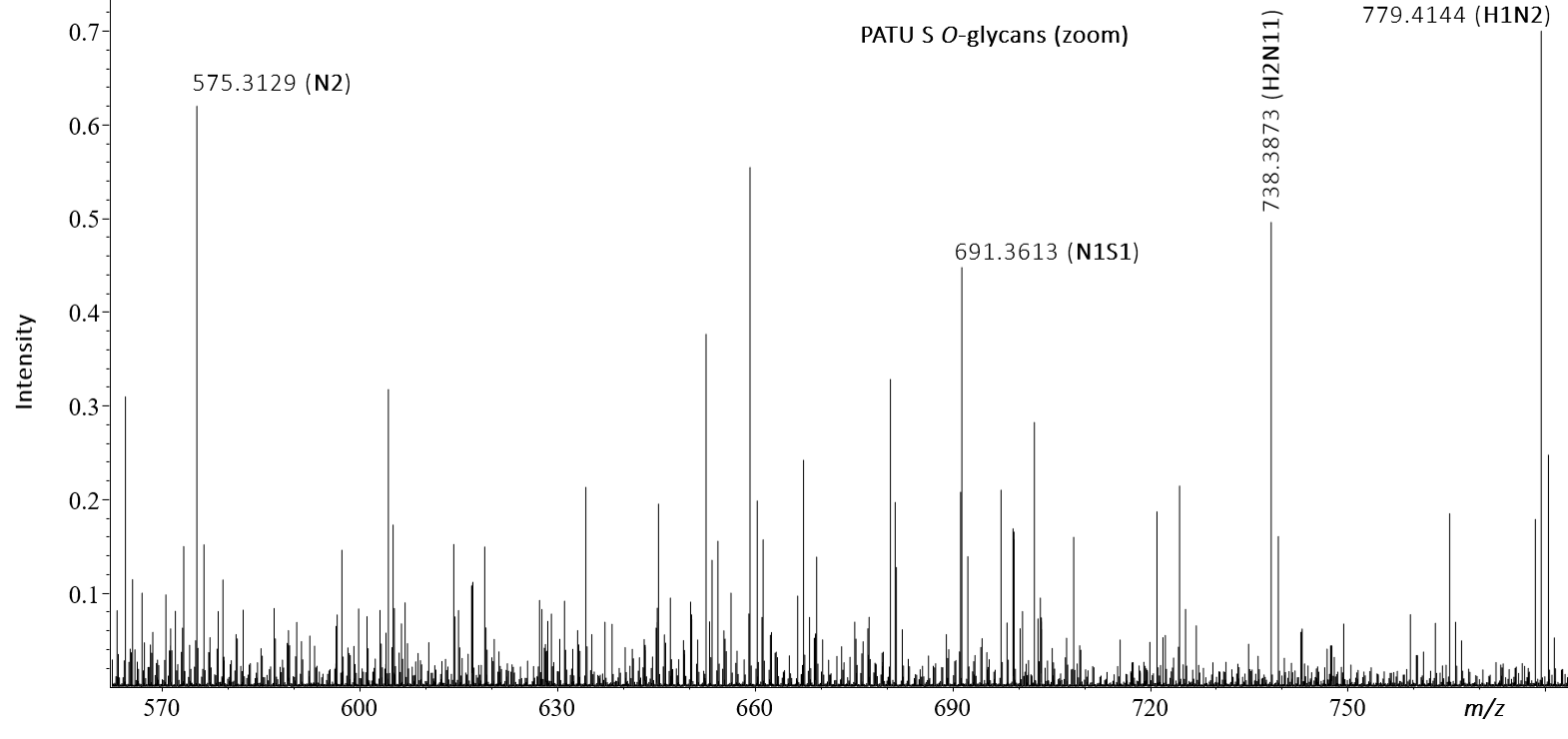


**Fig. 23.** Expanded view of the low mass region of MALDI-FTICR-MS spectra for PaTu S human pancreatic cancer cell line with annotated m/z values and compositions for detected *O*-glycans. Glycan compositions are given in the terms of hexose (H), N-acetylhexosamine (N), deoxyhexose (F), N-acetylneuraminic acid (S).


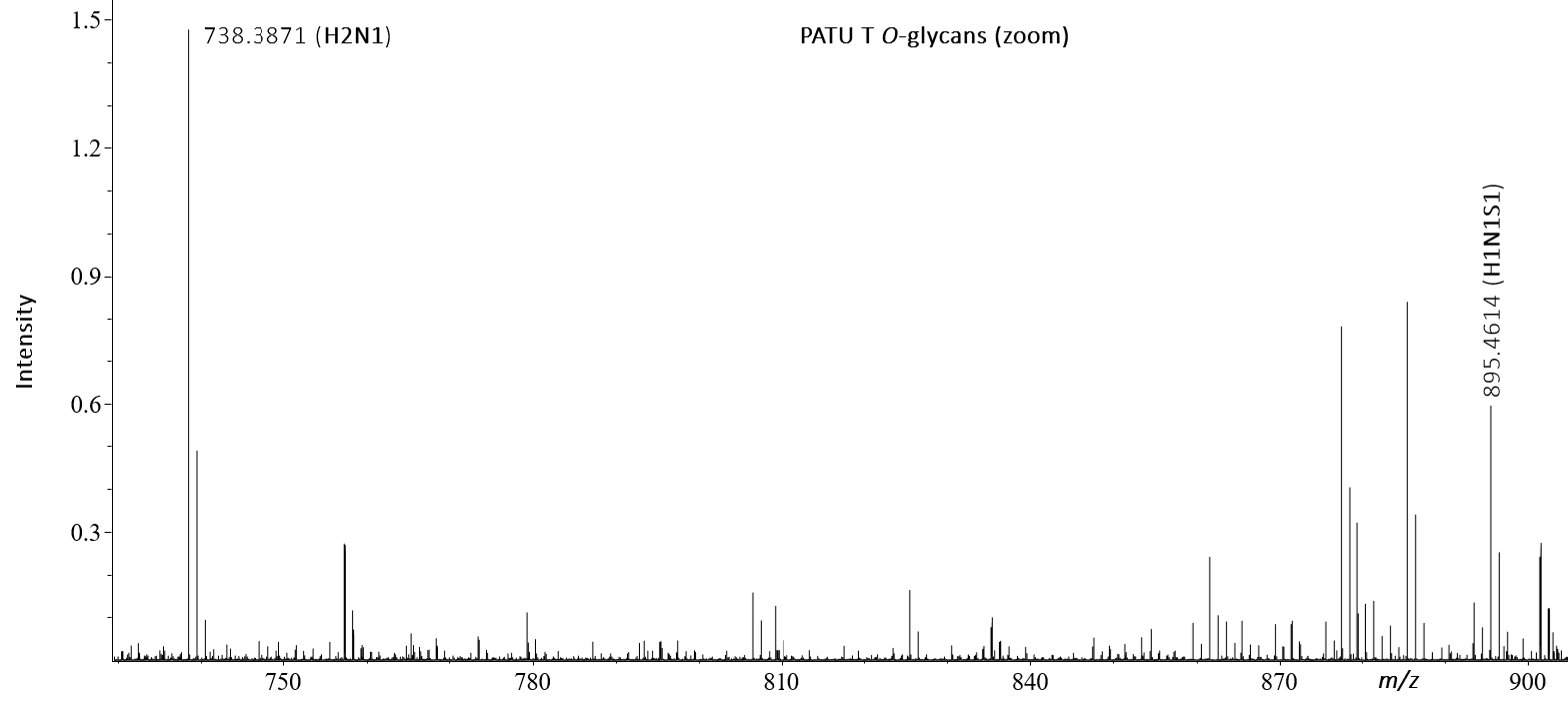


**Fig. 24.** Expanded view of the low mass region of MALDI-FTICR-MS spectra for PaTu T human pancreatic cancer cell line with annotated m/z values and compositions for detected *O*-glycans. Glycan compositions are given in the terms of hexose (H), N-acetylhexosamine (N), deoxyhexose (F), N-acetylneuraminic acid (S).


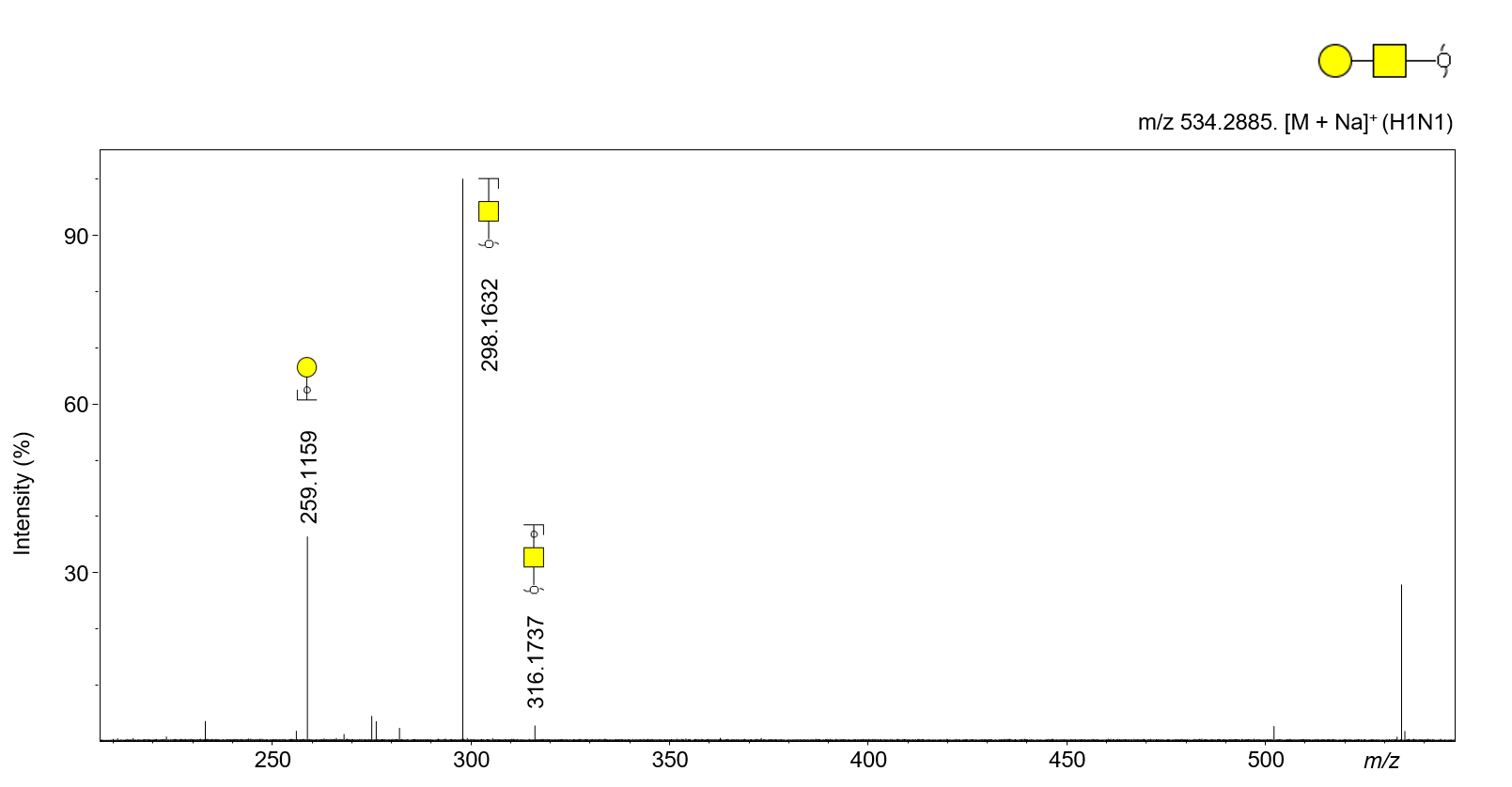


**Fig. 25.** Fragment ion spectra of precursor m/z 534.2885, [M + Na]^+^ from LS174T human colorectal cancer cell line *O*-glycans.


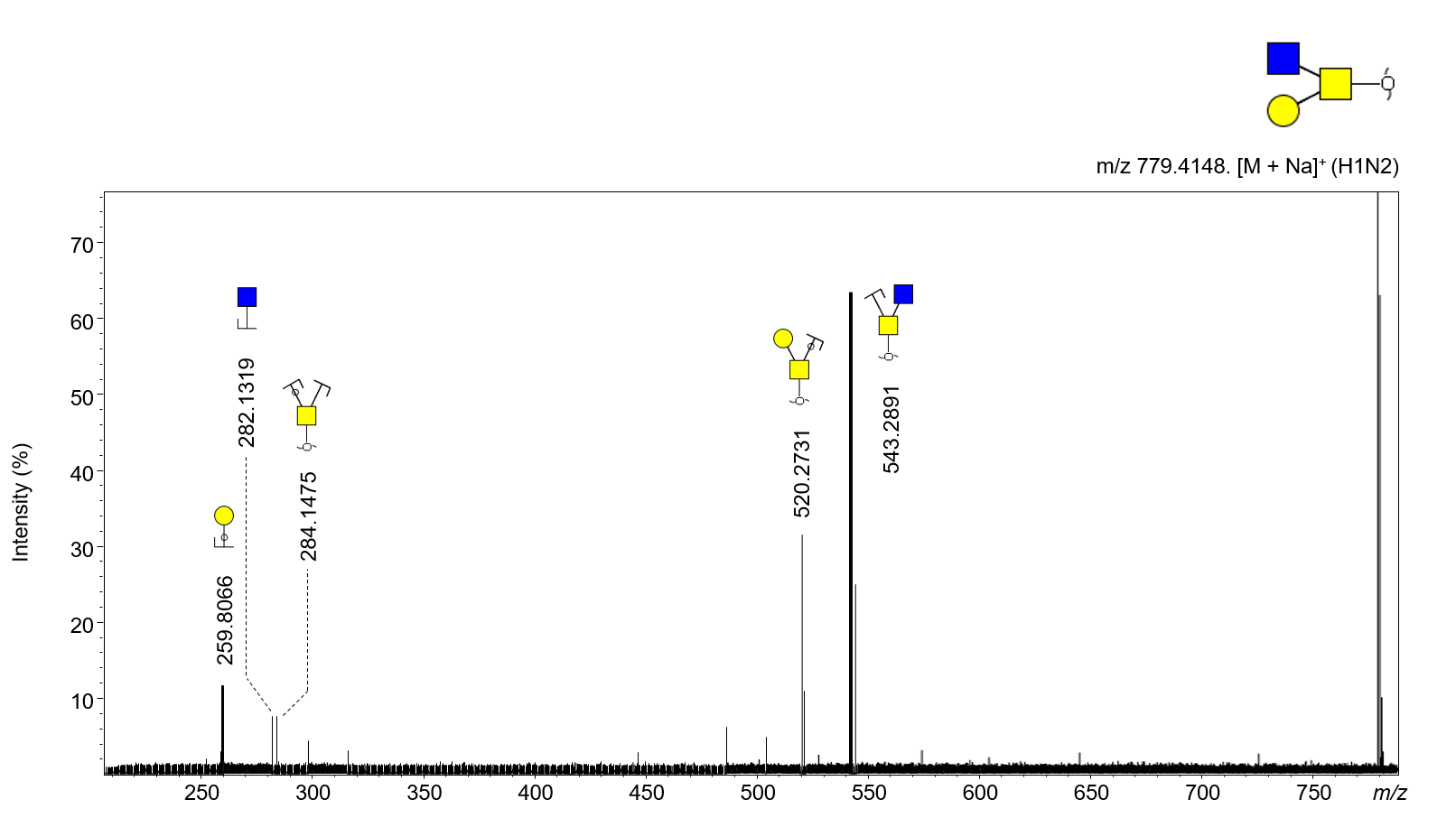


**Fig. 26.** Fragment ion spectra of precursor m/z 779.4148, [M + Na]^+^ from PaTu S human pancreatic cancer cell line *O*-glycans

References

[1] J.H.L. Chik, J. Zhou, E.S.X. Moh, R. Christopherson, S.J. Clarke, M.P. Molloy, N.H. Packer, Comprehensive glycomics comparison between colon cancer cell cultures and tumours: Implications for biomarker studies, J. Proteomics. 108 (2014) 146–162. https://doi.org/10.1016/j.jprot.2014.05.002.

[2] T. Zhang, I. van Die, B. Tefsen, S.J. van Vliet, L.C. Laan, J. Zhang, P. ten Dijke, M. Wuhrer, A.I. Belo, Differential O- and Glycosphingolipid Glycosylation in Human Pancreatic Adenocarcinoma Cells With Opposite Morphology and Metastatic Behavior, Front. Oncol. 10 (2020) 1–19. https://doi.org/10.3389/fonc.2020.00732.
